# Synthesis of *N*-peptide-6-amino-D-luciferin Conjugates

**DOI:** 10.3389/fchem.2018.00120

**Published:** 2018-04-19

**Authors:** Anita K. Kovács, Péter Hegyes, Gábor J. Szebeni, Lajos I. Nagy, László G. Puskás, Gábor K. Tóth

**Affiliations:** ^1^Department of Medical Chemistry, University of Szeged, Szeged, Hungary; ^2^Avidin Ltd., Szeged, Hungary; ^3^Department of Genetics, Biological Research Center, Hungarian Academy of Sciences, Szeged, Hungary

**Keywords:** bioluminescence, aminoluciferin, conjugate, protease activity, solid-phase peptide synthesis

## Abstract

A general strategy for the synthesis of *N*-peptide-6-amino-D-luciferin conjugates has been developed. The applicability of the strategy was demonstrated with the preparation of a known substrate, *N*-Z-Asp-Glu-Val-Asp-6-amino-D-luciferin (*N*-Z-DEVD-aLuc). *N*-Z-DEVD-aLuc was obtained via a hybrid liquid/solid phase synthesis method, in which the appropriately protected C-terminal amino acid was coupled to 6-amino-2-cyanobenzothiazole and the resulting conjugate was reacted with D-cysteine in order to get the protected amino acid-6-amino-D-luciferin conjugate, which was then attached to resin. The resulting loaded resin was used for the solid-phase synthesis of the desired *N*-peptide-6-amino-D-luciferin conjugate without difficulties, which was then attested with NMR spectroscopy and LC-MS, and successfully tested in a bioluminescent system.

## Introduction

In the recent years, numerous *in vivo* and *in vitro* analytical methods have been developed based on fluorescence and bioluminescence, including immunoassays, gene expression assays, bioimaging, investigation of infectious diseases etc., (Ioka et al., 2016; Kaskova et al., 2016); plate based, high-throughput viability assays addressing the detection of protease activity is in the focus of intensive research (Kepp et al., 2011). Protease activity can be detected with both fluorescent and bioluminescent detection systems, but with the latter the detection threshold is orders of magnitude lower than that of the fluorescent technique (O'Brien et al., 2005; Hickson et al., 2010; Gilbert and Boutros, 2016).

In the bioluminescent methods, diverse sets of luciferases and their substrates, luciferins have been applied in different cellular and animal models (Ioka et al., 2016; Kaskova et al., 2016). Aminoluciferin (aLuc) is a luciferin with its 6-position hydroxyl group substituted with an amino group. This modification allows aLuc to form amide bond with a peptide, while retaining the transport and bioluminescent properties of luciferin, resulting in a good substrate for different important proteases, which can be used for the determination of the enzymatic activity mentioned above (White et al., 1966).

**Figure 1:**
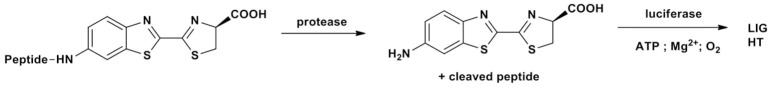
The operation of the bioluminescent system.

*N*-linked peptide-6-amino-D-luciferins can be substrates for different proteases, including metalloproteases, chymotrypsin-like, trypsin-like, and caspase-like proteases (O'Brien et al., 2008) They can be used for measuring protease enzyme activity in the following way: the protease enzyme to be measured recognizes the peptide part of the conjugate with the suitable peptide sequence, then cleaves the amide bond between the peptide and the aLuc, thus aLuc is released, which, in the presence of luciferase enzyme, emits light (Figure 1). The activity of the given protease enzyme can be determined from the amount of emitted light, as the emitted light is directly proportional to the activity of the enzyme (Leippe et al., 2011).

In the ongoing research the authors have developed and optimized a more efficient method for the synthesis of *N*-peptide-6-amino-D-luciferin conjugates in general, with a simpler set-up under milder conditions. *N*-Z-DEVD-aLuc was chosen to demonstrate the applicability of the strategy because it is a commercially available but very expensive compound, which is used to measure the activity of caspase-3, and consequently the efficiency of apoptosis-inducing drugs (Talanian et al., 1997; McStay et al., 2008).

### Literary overview

The logical method for the synthesis of *N*-peptide-6-amino-D-luciferin conjugates would start with the synthesis of the key molecule, 6-amino-2-cyanobenzothiazole. So far very few methods have been published for this step (Takakura et al., 2011; Gryshuk et al., 2013; McCutcheon et al., 2015; Hauser et al., 2016; Hsu et al., 2016; see Supplementary Table 1). The key steps of all these methods are the nitration, the cyanidation and the NO_2_ reduction; the methods differ in the starting material, the reagents, the solvents and the order of the transformations. Having examined these methods, it can be seen that they have disadvantages:

- A less optimal starting material may require an extra transformation during the synthesis (see Supplementary Table 2).
- Certain synthesis routes require too much reagents, some of which are expensive (see *Comments* in Supplementary Table 1).
- The use of an ill-chosen solvent leads to low yield during the chlorine-cyanide exchange (see Supplementary Table 3).
- A less optimal order of the transformations results in low yield (by transformation and, consequently, overall). With the optimal order, however, a transformation on one functional group does not result in a side reaction on the other functional group (see Supplementary Table 4).

Ideally, in the next step the amino group of the 6-amino-2-cyanobenzothiazole is blocked with a protecting group. However, the low nucleophilicity of the amino group makes its protection problematic, resulting in very low yield. Therefore, a different synthesis route is needed. One method has been published (Gryshuk et al., 2011; see Supplementary Table 5). Instead of a protecting group, the amino group of the 6-amino-2-cyanobenzothiazole is blocked with the protected C-terminal amino acid of the target sequence, and then the protecting group is removed from the C-terminal amino acid. In the following step, the remaining part of the target sequence is added and the side chain protecting groups of the peptide portion are removed; finally cysteine is added. However, the route has disadvantages:

- The mixed anhydride method for the acylation is not optimal, because it is not economical.
- Due to the basic conditions (pH 8) during the cysteine addition, there is a risk of racemization.
- The resulting materials are purified twice during the route, which is unnecessary.
- Yields were not determined, therefore it is difficult to evaluate the synthesis route.

## Materials and methods

### Materials

2-chlorobenzothiazole, HATU, TCFH, DCC, and D-Cys·HCl·H_2_O were obtained from AK Scientific Inc. (Union City, CA, USA). Z-Asp(OtBu)-OH and COMU were sourced from Bachem (Bubendorf, Switzerland). Fmoc-amino acids were purchased from Orpegen (Heidelberg, Germany) and Bachem (Bubendorf, Switzerland); Wang resin from Rapp Polymere GmbH (Tuebingen, Germany), TFFH from Fluorochem Ltd., (Hadfield, UK). The HOBt was sourced from Carbosynth Ltd (Compton, UK), trifluoroacetic acid gradient grade from VWR International (Radnor, PA, USA). The following reagents were purchased from Sigma-Aldrich (St. Louis, MO, USA): Deoxo-Fluor Reagent, PBS, trypsin, HEPES, CHAPS, DTT, EDTA, DMEM-F12, penicillin, streptomycin, 0.1% saponin. Alexa Fluor® 488 was bought from Thermo Fisher Scientific (Waltham, MA, USA). *N*-Z-DEVD-aLuc, C150 (Nagy et al., 2015; Hackler et al., 2016), and Ac-915 (Nagy et al., 2013) were synthesized by Avidin Ltd., (Szeged, Hungary).

Thin layer chromatography was performed on silica gel plates 60 F_254_ from Merck (Darmstadt, Germany). Melting points were determined using Büchi (Flawil, Switzerland) melting point apparatus Model B-545. pH values were measured with a Hanna HI 8424 pH meter. Cellulose extraction thimbles were purchased from Whatman (Maidstone, UK). Analytical reversed-phase high-performance liquid chromatography was performed on an Agilent 1,200 series separations module with diode array and multiple wavelength detector (Waldbronn, Germany), with a Luna C18(2) 100 Å column (10 μm, 250 × 4.6 mm) Phenomenex, (Torrance, CA, USA). The experiments were carried out at room temperature with a flow rate maintained at 1.2 ml min^−1^ at 220 nm wavelength (mobile phases solvent A: 0.1% TFA in Milli-Q water and solvent B: 0.1% TFA in AcN) using gradient elution. Separation was achieved on a Shimadzu (Kyoto, Japan) semi-preparative system with a Jupiter C18 300 Å column (10 μm, 250 × 21.20 mm), also from Phenomenex (mobile phases solvent A: 0.1% TFA in Milli-Q water and solvent B: 0.1% TFA in AcN) using gradient elution. Mass spectrometry data for the 2-chloro-6-nitrobenzothiazole (**1**) were collected on a Finnigan MAT TSQ 7,000 (Waltham, MA, USA) instrument, operating with APCI in negative ion mode, data for materials **2, 3, 8** were collected on Waters (Milford, MA, USA) SQ Detector with API mass spectrometer in positive ion mode; data for compounds **4** and **5** were recorded with Waters Q-Tof Premier Mass Spectrometer. ^1^H NMR and ^13^C NMR spectra were recorded using a Bruker DR × 500 spectrometer at 600 MHz and 150 MHz, respectively in [D6]DMSO. Chemical shifts were reported on the δ scale and *J* values were given in Hz.

Caspase-3 and the assay buffer were used from the Caspase-3 inhibitor drug screening kit from BioVision (Milpitas, CA, USA). Black plastic microtiter plates were purchased from Tomtec (Budapest, Hungary), Z-VAD-fmk from Calbiochem (San Diego, CA, USA) and Merck Millipore, (Billerica, MA, USA), luminescence detection reagent from Promega (Madison, WI, USA). 9661S caspase-3 antibody from Cell Signaling Technology (Leiden, The Netherlands). Luminescence was recorded as counts per seconds by a plate reader Perkin Elmer Wallac VICTOR 1420 (Waltham, MA, USA). A549 non-small cell lung carcinoma cells were purchased from the ATCC (Manassas, VA, USA) and U87-Luc glioblastoma cell line from Perkin Elmer (Waltham, MA, USA). Tissue culture dishes (60 mm dishes. 96-well plates) were purchased from Corning (Corning, NY, USA). DMEM and FBS were purchased from Gibco BRL (Gaithersburg, MD, USA). Male SCID mice (6 weeks old, 22–24 g body weight) were supplied by Innovo Ltd. (Budapest, Hungary). We used IVIS 100 imaging instrument from Xenogen, (Alameda, CA, USA). CellQuest™ software was bought from Becton Dickinson (Franklin Lakes, NJ, USA), GraphPad Prism® 5 from GraphPad Software (La Jolla, CA, USA).

The mouse studies were performed according to the Institutional and National Animal Experimentation and Ethics Guidelines in possession of an ethical clearance (XXIX./3610/2012), provided by the Head of Foodchain-safety and Animal Health of the Csongrad County Government Office. Document number: CSI/01/126/2013. Valid until 8th of January 2018.

### Methods

#### Preparation of 2-chloro-6-nitrobenzothiazole (1)

Four hundred and thirty eight milliliter cc H_2_SO_4_ was cooled to 10°C in a 2-liter triple-neck round-bottomed flask. 100 g (0.59 mol) 2-chlorobenzothiazole was dripped to the sulfuric acid over a period of 2 h, meanwhile the reaction mixture was stirred vigorously and the temperature was held under 15°C. Sixty-Six grams (0.66 mol) powdered KNO_3_ was added to the reaction mixture in small quantities in 45 min, the temperature was still kept under 15°C. Then the reaction mixture was allowed to warm up to room temperature, and stirring was continued at room temperature for 2 h. It was poured into 4 liters of ice and water. Yellow precipitation formed, which was filtered and washed until the pH of the filtrate became neutral. The crystalline compound was dried at room temperature, followed by its recrystallization from ethyl acetate in order to get rid of 2-chloro-5-nitrobenzothiazole as the single side product. The resulting material was a pale yellow crystal, its weight was 104.90 g (0.49 mol), yield 83%, mp 191-192°C (EtOAc), (lit. mp 190–191°C, Katz, 1951). ^1^H-NMR (CDCl_3_, 500 MHz) δ 8.77 (s, ^1^H), 8.41 (d, *J* = 9.0 Hz, ^1^H), 8.10 (d, *J* = 9.0 Hz, ^1^H) (Supplementary Figure 1). The spectral data matched that in the literature (Shinde et al., 2006) *m/z* (TSQ): 213.93 [M-H]^−^ (Supplementary Figure 2), RP-HPLC: 70–100% B in 15 min + 100% B in 5 min, *t*_*R*_ = 7.899 min (Supplementary Figure 3), TLC: *n*-hexane/dioxane = 2:1; R_f_: 0.42.

#### Preparation of 6-amino-2-chlorobenzothiazole (2)

Twenty-five gram (0.12 mol) 2-chloro-6-nitrobenzothiazole (**1**) packed in a paper cup and 500 ml ethyl acetate, 30 g NH_4_Cl (0.56 mol), 200 ml water, and 20 g reduced Fe powder in a 1-liter round-bottomed flask was put in a Soxhlet apparatus and heated under reflux for 8 h while continuously stirring the mixture. This way, the continuous dissolution of the starting material, which has low solubility in ethyl acetate, was ensured, allowing for unmonitored and unmanaged operation while we could efficiently recycle a small amount of ethyl acetate to dissolve a larger amount of 2-chloro-6nitro-benzothiazole, thus making the procedure more economical. In order to get rid of the remaining water/NH_4_Cl/Fe-powder as lower part, the upper part ethyl acetate layer was decanted, and this process was repeated twice with 100 ml ethyl acetate, respectively. Decantation was employed instead of using a separatory funnel because the lower aqueous phase too viscous. We had no iron waste as in our method no chemical transformation of the iron occurred, the iron was 100% recyclable: the iron powder was filtered off, and then washed on a Büchner funnel with distilled water. The combined organic phase was dried over anhydrous Na_2_SO_4_, filtered and evaporated on rotary evaporator. The resulting material was a yellow crystal, its weight was 18.90 g (0.10 mol), yield 88%, mp 154–156°C (EtOAc) (lit.155–157°C, Katz, 1951). ^1^H NMR ([D6]DMSO, 600 MHz) δ (d, *J* = 8.4 Hz, ^1^H), 7.04 (d, *J* = 2.4 Hz, ^1^H), 6.78 (dd, *J*_1_ = 1.8 Hz, *J*_2_ = 8.4 Hz, ^1^H), 5.53 (bs, ^2^H) (Supplementary Figure 4), ^13^C NMR ([D6]DMSO, 150 MHz) δ 148.24, 145.51, 142.00, 137.86, 123.13, 115.51, 104.18 (Supplementary Figure 5). The spectral data matched that in the literature (Reddy et al., 2010). *m/z* (ESI): 185.0 [M + H]^+^ (Supplementary Figure 6), RP-HPLC: 5–80% B in 25 min + 3 min up to 100% B + 100% B in 5 min, *t*_*R*_ = 11.527 min (Supplementary Figure 7), TLC: *n*-hexane/dioxane = 2:1; R_f_: 0.76.

#### Preparation of 6-amino-2-cyanobenzothiazole (3)

In order to get a suspension, 6.1 g (93.0 mmol) KCN was sonicated in 400 ml DMAA for 3 × 15 min. The suspension was heated in an oil-bath at 98–100°C under argon atmosphere and then 6.86 g (37.15 mmol) 6-amino-2-chlorobenzothiazole (**2**), dissolved in 20 ml DMAA, was dripped to this reaction mixture over a period of 50 min. This resulting mixture was heated in an oil-bath at 110°C and stirred continuously under argon atmosphere for 12 h. After 12 h stirring there was still starting material in the mixture. An increased conversion from the starting ratio of 2.5:1 for KCN/6-amino-2-chlorobenzothiazole (**2**) to the ratio of 3.4:1 was achieved by adding 2.20 g (33.80 mmol) of KCN. This was followed by 5 h stirring under the conditions described above, and after that procedure the remaining amount of 6-amino-2-chlorobenzothiazole (**2**) was insignificant. The reaction mixture was poured on a mixture of 200 g ice, 400 ml 1 M KH_2_PO_4_ and 300 ml ether. The organic phase was separated from the aqueous phase. The latter was extracted with 2 × 250 ml ether, then with 2 × 200 ml ethyl acetate. The combined organic phases were washed with 2 × 300 ml water and 1 × 300 ml brine, dried over anhydrous Na_2_SO_4_ and concentrated on rotary evaporator. The resulting material was a pale brown solid, its weight was 6.4 g (crude). The material was recrystallized from acetone, and the impurities were removed by adding activated charcoal to the solution. The weight of the desired purified material was 5.09 g (29.10 mmol), yield 78%, mp 218–219°C (EtOAc) (lit. mp 216–218°C, White et al., 1966). ^1^H NMR ([D6]DMSO, 600 MHz) δ (d, *J* = 9.0 Hz, ^1^H), 7.22 (d, *J* = 1.8 Hz, ^1^H), 7.01 (dd, *J*_1_ = 1.8 Hz, *J*_2_ = 9.0 Hz, ^1^H), 4.40 (bs, ^2^H) (Supplementary Figure 8), ^13^C NMR (150 MHz, ([D6]DMSO) δ 150.06, 144.10, 138.75, 128.86, 125.62, 118.19, 114.71, 103.54 (Supplementary Figure 9). The spectral data matched that in the literature (McCutcheon et al., 2015). *m/z* (ESI) 176.0 [M + H]^+^ (Supplementary Figure 10), RP-HPLC: 5–80% B in 25 + 3 min up to 100% B + 5 in 100% B, *t*_*R*_ = 16.692 min (Supplementary Figure 11), TLC: *n*-hexane/dioxane = 2:1; R_f_: 0.48. The remaining KCN was reacted with KH_2_PO_4_ in order to get non-toxic KOCN.

#### Preparation of Fmoc-Asp(OtBu)-6-amino-2-cyanobenzothiazole (4)

6.30 g (15.30 mmol, 1.5 equiv) Fmoc-Asp(OtBu)-OH, which was previously dried in a vacuum desiccator, and 4.30 g (15.30 mmol, 1.5 equiv) TCFH were solved in 35 ml dry DCM. The mixture was stirred for 60 min at room temperature. First 3.06 ml (18.36 mmol, 1.8 equiv) DIPEA, then 1.79 g (10.20 mmol, 1 equiv) 6-amino-2-cyanobenzothiazole, which was previously dried in a vacuum desiccator, were added. Further 200 ml dry DCM was added to get complete dissolution of the materials. After stirring the reaction mixture overnight at room temperature, it was transferred into a separatory funnel and washed with water (2 × 30 ml), with saturated NaHCO_3_-solution (2 × 30 ml), then with water again (2 × 30 ml), and finally with brine (2 × 30 ml). It was dried over anhydrous Na_2_SO_4_, finally concentrated on rotary evaporator. The resulting crude material was a yellowish-brown powder, its weight was 6.17 g. RP-HPLC analysis showed a yield of 73%. *m/z* (TOF) 569.2070 [M + H]^+^ (Supplementary Figure 12), RP-HPLC (for the purified compound): 50–100% B in 25 min, t_R_ = 10.591 min (Supplementary Figure 13), TLC: ethanol (EtOH)/toluene 50:7.5; Rf: 0.58.

#### Synthesis of Fmoc-Asp(OtBu)-6-amino-D-luciferin (5)

2.96 g (5.20 mmol) Fmoc-Asp(OtBu-)-6-amino-2-cyanobenzothiazole was dissolved in the mixture of 35 ml MeOH and 20 ml THF. 1.37 g (7.80 mmol) D-cysteine·HCl·H_2_O, dissolved in 10 ml distilled water, was added to the mixture at room temperature under argon atmosphere, while stirring continuously under pH control (starting pH: 1.67). After 20 min stirring at room temperature 16 ml 5% (m/m) NaHCO_3_ was added dropwise over a period of 1 h to the mixture in order to release cysteine from its salt while continuously checking pH. Reaching pH 2.5, a fine, yellow solid material, Fmoc-Asp(OtBu)-6-amino-D-luciferin free carboxylic acid, started to precipitate. At pH 6.1, this material started to dissolve, and at pH 7.36, it dissolved completely. Here the Fmoc-Asp(OtBu)-6-amino-D-luciferin formed Na-salt, which dissolved under the basic conditions. After an additional 20 min stirring at room temperature the organic solvent was removed under reduced pressure. Water and methanol forms an azeotrope, and the two solvents were therefore removed together through the distillation. Due to the decrease in the concentration of the water, from the remaining aqueous solution a yellow solid material, Fmoc-Asp(OtBu)-6-amino-D-luciferin Na-salt, precipitated. This, however, was just an irrelevant event during the process, as our goal was the removal of the methanol. The aforementioned precipitate is water soluble, so then it was dissolved again in 20 ml water and extracted with 1 × 15 ml ethyl acetate in order to get rid of possible impurities. Having dropped this solution on a mixture of ice and glacial acetic acid (adjusted to pH 3), a fine yellow precipitate formed, Fmoc-Asp(OtBu)-6-amino-D-luciferin free carboxylic acid. It was allowed to settle for 10 min, filtered and washed with 3 × 10 ml water, then air-dried to constant weight, which was 2.83 g (4.20 mmol), yield 81%. *m/z* (TOF) 673.1882 [M + H]^+^ (Supplementary Figure 14), RP-HPLC: 50–100% B in 25 min, *t*_*R*_ = 21.046 min (Supplementary Figure 15), TLC: toluene/EtOH 50:30 saturated with water, R_f_: 0.58.

#### Attachment of Fmoc-Asp(OtBu)-6-amino-D-luciferin to solid support (6)

Solid phase peptide synthesis was performed manually by using a solid phase vessel attached to a rotating apparatus. 0.127 g (0.10 mmol, 1 equiv) p-alkoxybenzyl alcohol resin was allowed to swell in anh DCM for 20 min. After the removal of the DCM, 0.202 g (0.30 mmol, 3 equiv) Fmoc-Asp(OtBu)-amino-D-luciferin, 0.062 g (0.30 mmol, 3 equiv) DCC, 0.041 g (0.30 mmol, 3 equiv) HOBt, and 0.037 g (0.10 mmol, 1 equiv) DMAP, dissolved in 10 ml anh DCM, was added to the resin. The coupling reaction was shaken for 3 h at room temperature. After the removal of the coupling mixture, the resin was rinsed with DCM (3 × 10 ml), MeOH (1 × 10 ml) and then with DCM (3 × 10 ml) again. The coupling reaction was repeated with the soln of 0.067 g (0.10 mmol, 1 equiv) Fmoc-Asp(OtBu)-amino-D-luciferin, 0.062 g (0.30 mmol, 3 equiv) DCC, 0.041 g (0.30 mmol, 3 equiv) HOBt and 0.037 g (0.10 mmol, 1 equiv) DMAP in 5 ml anh DCM at room temperature for 2 h. The resin was drained and rinsed with DCM (3 × 10 ml), MeOH (1 × 10 ml), DCM (3 × 10 ml), then dried to constant weight.

##### Determination of load

5 mg of dried loaded resin was treated with a mixture of TFA/water (500 μl, with the ratio of 95:5) for 1 h at room temperature. This was followed by the addition of 500 μl water to the cocktail, which was then filtered off. 10 μl from the filtrate was injected to analytical RP-HPLC and the area of the Fmoc-Asp-6-amino-D-luciferin on the resulted chromatogram was compared with the area of 10 ul Fmoc-Asp-6-amino-D-luciferin stock solution with the concentration of 1 mg/ml. The resulting load was 47.8%.

#### Preparation of *N*-Z-DEVD-aLuc (8)

##### Fmoc deprotection

Fmoc deprotection was carried out by suspending the resin in 20% (v/v) piperidine/DMF (5 ml) and agitating the vessel at room temperature for 2 × 10 min. The suspension was then filtered and the resin was washed with DMF (3 × 5 ml), MeOH (3 × 5 ml), DMF (3 × 5 ml).

##### SPPS peptide coupling (7)

Fmoc-Val-OH (3 equiv), DCC (3 equiv), and HOBt (3 equiv) dissolved in DMF were added to the previously swollen and Fmoc-deprotected loaded resin (1 equiv). The resulting suspension was agitated at room temperature for 2 h and the resin was then rinsed with DMF (3 × 5 ml), MeOH (3 × 5 ml), DMF (3 × 5 ml).

The same procedure was carried out with Fmoc-Glu(OtBu)-OH (3 equiv) and Z-Asp(OtBu)-OH, (3 equiv). The presence or absence of the *N*α-free amino group was monitored using the Kaiser test.

##### Cleavage of peptide from the resin

The peptide-resin (**7**) was treated with a solution of TFA/water (95:5 v/v) for 2 h at room temperature. After the removal of the cleaving mixture, the resin was rinsed with AcN (3 × 10 ml), MeOH (1 × 10 ml) and with AcN (3 × 10 ml) again. The resulting material is a yellow liquid, which was lyophilized afterwards. RP-HPLC for the crude compound: 5–80% B in 25 min + 3 min up to 100% B + 5 min in 100% B, t_R_ = 18.483 min (Supplementary Figure 16).

##### Purification of crude peptide

**Figure 2:**
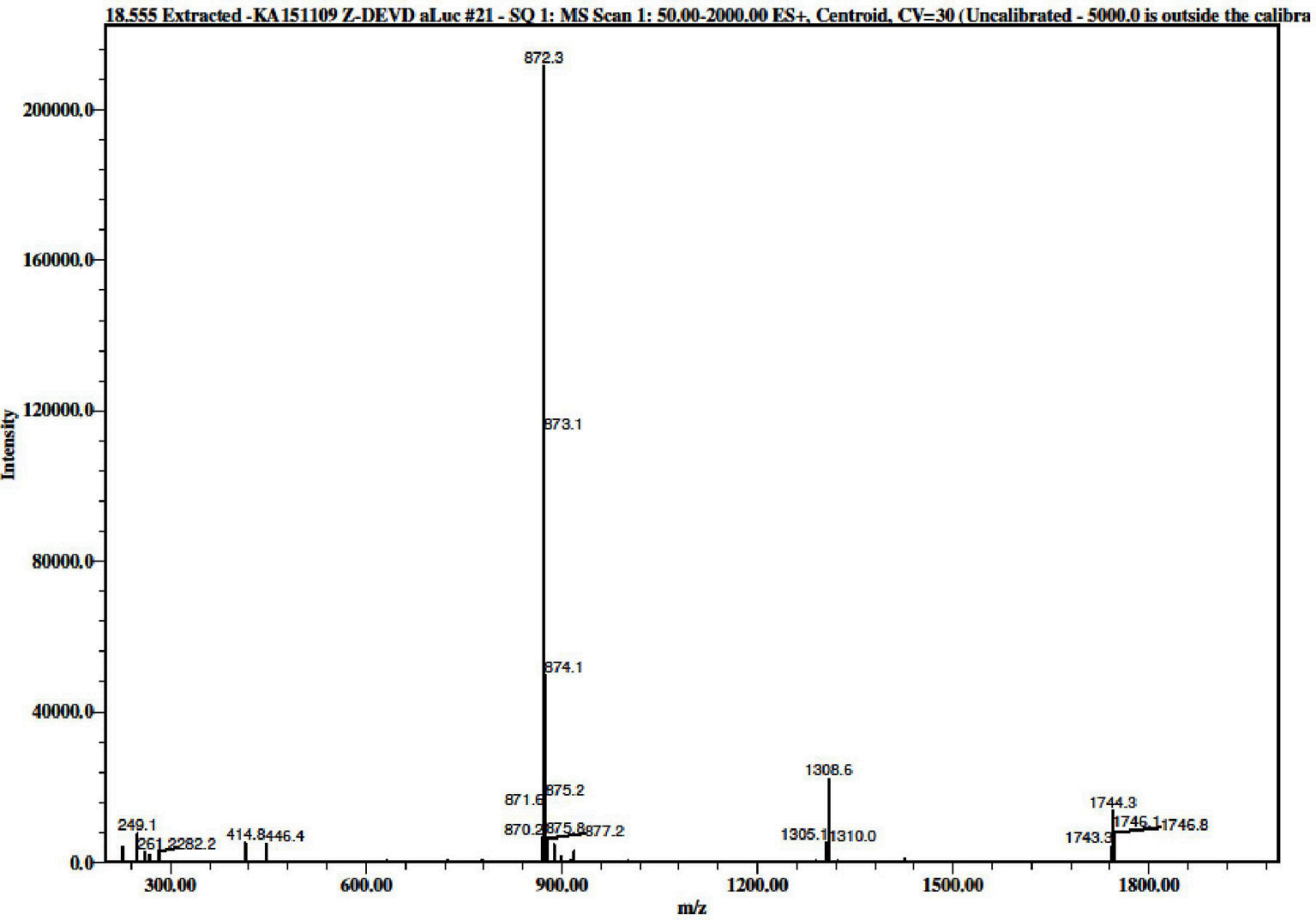
Mass spectrum (ESI) of the purified *N*-Z-DEVD-aLuc **(8)** 872.3 = [M+H]^+^.

**Figure 3:**
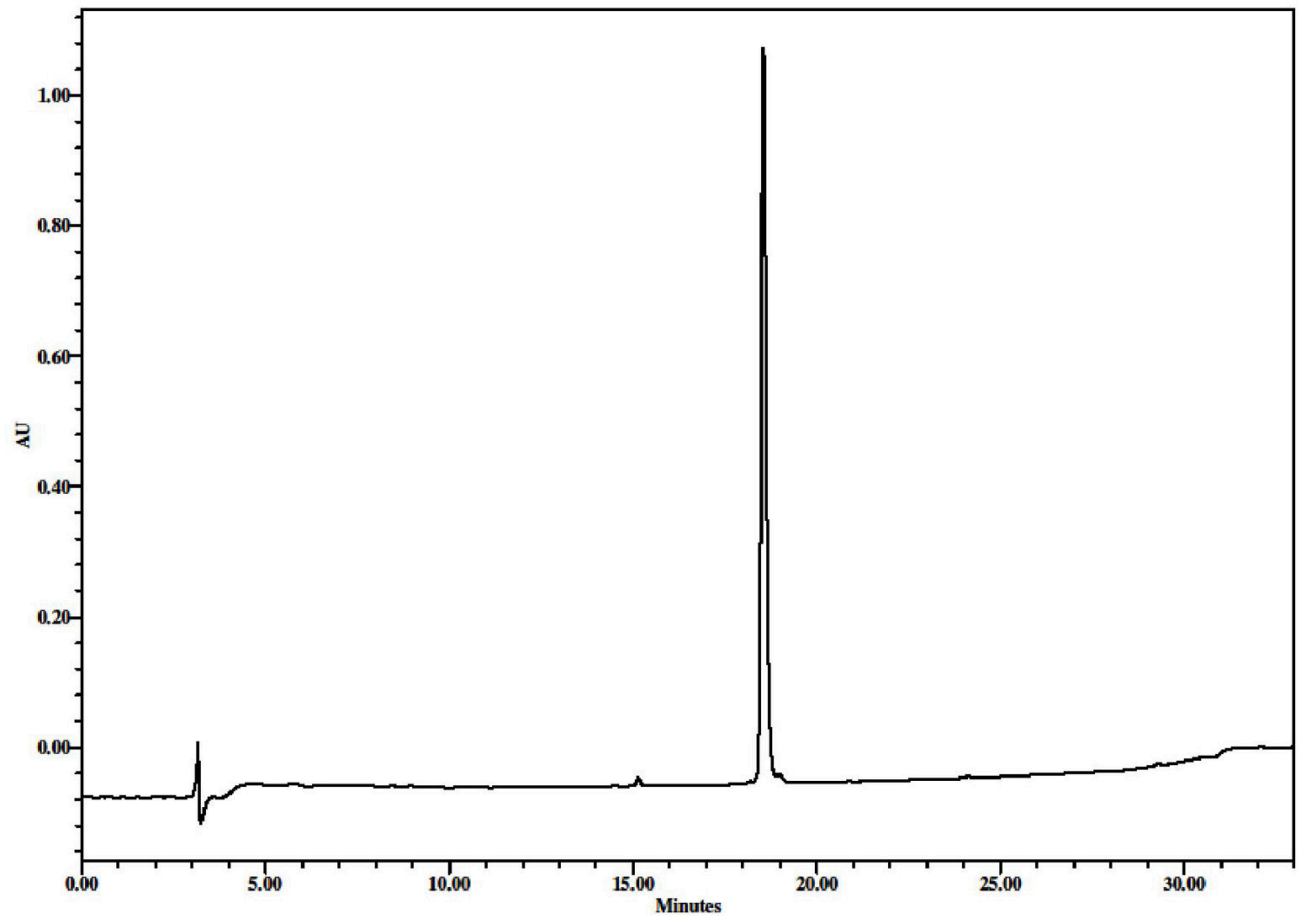
RP-HPLC profile of the purified *N*-Z-DEVD-aLuc **(8)**, 5–80% B in 25 + 3 min up to 100% B + 5 min in 100% B, *t*_*R*_ = 18.555 min.

26 mg crude peptide was dissolved in acetic acid/water (1.5 ml, with the ratio of 1:1), then filtered, using a 0.45 μm nylon filter. Gradient elution was used, 0–60% eluent B in 60 min at a 3 ml min^−1^ flow rate with detection at 220 nm. Pure fractions were collected and lyophilized to give a pale yellow material, the weight of which was 11.4 mg (0.013 mmol). ^1^H NMR (600 MHz, [D6]DMSO) δ 10.26 (bs, ^1^H), 8.61 (s, ^1^H), 8.42 (d, *J* = 7.2 Hz, ^1^H), 8.08 (d, *J* = 9.0 Hz, ^2^H), 7.81 (bs, ^1^H), 7.64 (dd, *J*_1_ = 9.0 Hz, *J*_2_ = 29.4 Hz, ^2^H), 7.34 (s, ^5^H), 5.42 (t, *J* = 8.4 Hz, ^1^H), 5.02 (s, ^2^H), 4.69 (d, *J* = 7.2 Hz, ^1^H), 4.34 (dd, *J*_1_ = 5.4 Hz, *J*_2_ = 29.4 Hz, ^2^H), 4.12 (bs, ^1^H), 3.77 (t, *J* = 10.8 Hz, ^1^H), 3.68 (dd, *J*_1_ = 8.4 Hz, *J*_2_ = 11.4 Hz, ^1^H), 2.77 (bs, ^1^H), 2.61–2.68 (m, ^2^H), 2.25–2.46 (m, ^3^H), 1.76–1.99 (m, ^2^H), 0.83–0.86 (m, ^7^H) (Supplementary Figure 17 part 1,2,3), ^13^C NMR (150 MHz, [D6]DMSO) δ 174.54, 172.00, 171.61, 171.38, 170.24, 159.62, 156.32, 149.14, 138.66, 137.31, 136.71, 128.83, 128.28, 128.19, 124.65, 120.28, 112.04, 78.64, 66.01, 58.21, 52.53, 51.86, 51.24, 36.77, 36.31, 35.23, 30.98, 30.52, 27.59, 19.53 (Supplementary Figure 18 part 1,2), *m/z* (ESI) 872.3 [M + H]^+^ (Figure 2), RP-HPLC: 5–80% B in 25 min + 3 min up to 100% B + 5 min in 100% B, *t*_*R*_ = 18.555 min (Figure 3).

#### *N*-Z-DEVD-aLuc biochemical assay

Caspase-3 and the assay buffer were used from the caspase-3 inhibitor drug screening kit. Caspase-3 was used in ten-fold serial dilution starting from 227 mU to 22.7 μU/reaction. *N*-Z-DEVD-aLuc substrate was applied in 100 μM to 1 μM in 25 μl final reaction volume in a black plastic microtiter plate. The effect of the pan-caspase inhibitor Z-VAD-fmk was tested in equimolar ratio of *N*-Z-DEVD-aLuc at 10 μM with 2.27 mU/reaction caspase-3. After 45 min incubation at 37°C we added 25 μl luminescence detection reagent to each well. Luminescence was recorded as cps by a plate reader within 5 min. Blank wells contained each component except caspase-3. Presented values are blank-subtracted.

#### Cell lines and culture

A549 cells and U87-Luc glioblastoma cells were maintained in DMEM-F12 and DMEM cell culture media, respectively. Both type of medium were supplemented with 10% (v/v) heat-inactivated FBS, 100 units/ml penicillin and 100 mg/ml streptomycin at 37°C in a humidified atmosphere containing 5% CO_2_.

#### *N*-Z-DEVD-aLuc cellular assay

A549 non-small cell lung carcinoma cells (2 × 10^6^) were plated in 60 mm dishes in DMEM-F12 media. After cell attachment (24 h), the cells were treated with curcumin analog C150 (5 μM to 1.25 μM) in order to induce apoptosis in 5 ml final volume (Nagy et al., 2015). After 24 h incubation supernatant was harvested and kept on ice. Cells were washed with PBS and trypsinized (5 min, 37°C). Supernatant, washing PBS and media blocked trypsin were mixed and centrifuged down (5 min, 4°C, 1,800 g). Lysis buffer was diluted with distilled water (five times concentrated lysis buffer: 250 mM HEPES, pH 7.4, 25 mM CHAPS, 25 mM DTT, and 50 μl 1x concentration lysis buffer was added to the pellet, resuspended and kept on ice for 15 min. Samples were centrifuged down for 10 min (4°C, 11,000 g). Supernatant of the lysate was harvested and kept on ice for analysis. *N*-Z-DEVD-aLuc was dissolved in DMSO at 10 mM and used in 10 μM in the assay. Assay buffer was diluted in distilled water (ten times concentrated assay buffer: 200 mM HEPES, pH 7.4, 1% (V/V) CHAPS, 50 mM DTT, 20 mM EDTA, 95 μl 1x concentration assay buffer containing 10 μM *N*-Z-DEVD-aLuc was measured in 96-well tissue culture plate and 5 μl lysate was assayed in triplicates to detect luminescence proportional to caspase-3 activity. Blank contained 5 μl assay buffer instead of the lysate. After 4 h reaction 50 μl reaction mixture was measured into black plastic microtiter plate and 50 μl luminescent detection reagent was added. Luminescence was recorded as cps by a plate reader within 5 min. Presented values are blank-subtracted.

#### *In vivo* animal model

Male SCID mice (6 weeks old, 22–24 g body weight) were housed in sterile cages at Avidin Ltd. The mice were fed autoclaved food and sterile water *ad libitum*. For inoculation, the U87-Luc cells were trypsinized, washed and resuspended in sterile PBS. The mice were injected subcutaneously with this suspension (3 × 10^6^ cells in 0.2 ml), in the dorsal region, unilaterally. All operative procedures and animal care conformed strictly to the Hungarian Council on Animal Care guidelines. (Approval provided by Head of Foodchain-safety and Animal Health of the Csongrad County Government Office. Document number: CSI/01/126/2013; valid until 8th of January 2018.) 18 days after inoculation the mice were treated with Ac-915, a lipid droplet binding thalidomide analog inducing oxidative stress and apoptosis in glioblastoma cells (Nagy et al., 2013). Ac-915 was dissolved in DMSO:solutol 3:1 mixture, then diluted in PBS four times and injected i.p. at a 20 mg/kg dose except negative control mice which were injected by PBS only. Six hours after drug administration, to monitor apoptosis induction, the mice were injected i.p., with 50 mg/kg *N*-Z-DEVD-aLuc in PBS, followed by anesthetization in 2–3% isoflurane atmosphere. 30 min after the injection of the substrate, the mice were imaged using a charge coupled device camera in the IVIS 100 imaging instrument.

#### Statistics

Statistical significance was calculated with unpaired *t*-test (two-tailed, homoscedastic) between untreated and one treated sample. Each point represents the average of 3 wells ± SEM. Values are blank-subtracted (blank = no caspase). ^*^p < 0.05; ^**^p < 0.01; ^***^p < 0.001.

## Results and discussion

**Figure 4:**
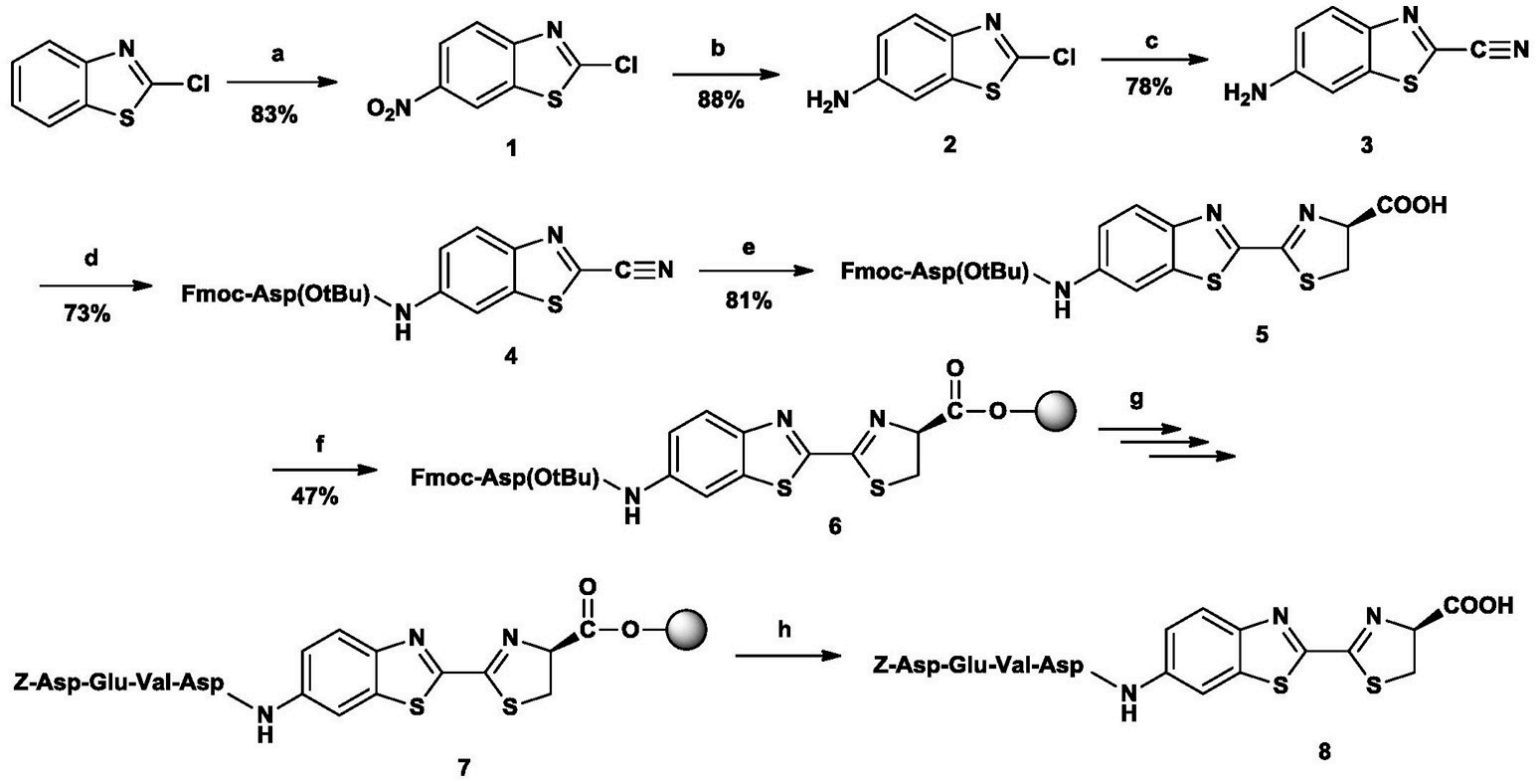
The synthetic route to *N*-Z-DEVD-aLuc. Reagents and conditions: (a) ccH_2_SO_4_/KNO_3_, 0–15°C, 5h (b) EtOAc, NH_4_Cl, H_2_O, Fe powder, reflux, 8h (c) KCN, DMAA, 110°C, 12h (d) Fmoc-Asp(OtBu)-OH, TCFH, dry DCM, DIPEA, overnight, rt (e) D-Cys Hcl H_2_O, THF, MeOH, H_2_O, 5 m/m% NaHCO_3_, 2h, rt (f) p-alkoxybenzyl alcohol resin, dry DCM, DCC, HOBt, DMAP, 5 h 30 min (g) Fmoc -/Z-protected amino acid. DCC. HOBt, DMF, 2 h, rt; 20% (v/v) piperidine for Fmoc deprotection, 20 min, rt (h) TFA/H_2_O 95:5 (v/v), 2 h, rt.

The desired peptide-luciferin conjugate (*N*-Z-DEVD-aLuc) was reached in an 8-step route (Figure 4, Supplementary Table 6):

nitration → reduction → chlorine-cyanide exchange → attachment of the C-terminal amino acid of the target sequence → cysteine addition → attachment to resin → solid-phase peptide synthesis → cleavage from resin.

As starting material, cheap, commercially available 2-chlorobenzothiazole was used, which was first nitrated with a mixture of potassium nitrate and concentrated sulphuric acid, keeping the temperature at 0°C (Katz, 1951). The structure of the resulting 2-chloro-6-nitrobenzothiazole (**1**) was attested by ^1^H-NMR spectrum. The next step was the reduction of the nitro-group. First the mixture of ethanol/glacial acetic acid/iron powder was used (Katz, 1951). Although the reaction was successful, the resulting by-product (iron(III) acetate) was difficult to dispose of, so this method was dismissed. When trying the reduction with tin(II) chloride/glacial acetic acid/concentrated hydrogen chloride, large amounts of by-product formed, in which the chlorine was split off or substituted with a hydroxyl group, so this method also had to be dismissed. The third possibility was the application of sodium pyrosulphite, which also turned out to be unsatisfactory due to the reduction giving a very low yield. Using zinc/hydrogen chloride led to the same results as with tin(II) chloride.

Finally, applying ethyl acetate, water, ammonium chloride, and iron powder system solved the problem and the reduction was successful with a good yield. Using a Soxhlet extractor turned out to be a solvent-sparing, thus environmentally-friendly method and processing the obtained product was also simple: the solution had to be decanted in order to get rid of the iron powder and then extracted.

The next step—the chlorine/nitrile exchange in the 6-amino-2-chlorobenzothiazole (**2**)—is the key in the production of the desired conjugates. Six different methods had to be tried, the common features of which were the polar aprotic non-aqueous solvent, the high temperature and the long reaction time (a) anh DMSO/KCN, 160°C, 10 h; (b) anh DMSO/KCN/KI, 160°C, 10 h; (c) anh DMSO/18-crown-6/KCN, 120°C, 8 h; (d) anh DMF/KCN, 140°C, 12 h; (e) anh HMPA/KCN, 140°C, 10 h; (f) *f)* anh DMAA/KCN/KI, 120°C, 8 h).

The first five methods had to be dismissed due to the low yield (15–20%). When trying DMAA, however, it turned out that KCN is dissolved best in this solvent, resulting in relatively high yield (75–80%) This means that the success of the chlorine/nitrile exchange depends on the rate of KCN dissolution.

An Fmoc-protected amino acid (Fmoc-Asp(OtBu)-OH) was coupled to the 6-amino-2-cyanobenzothiazole (**3**). As, due to the deactivated amino group, the amide bond could not be formed with the usual coupling reagent, DCC, a much more activating coupling agent was necessary. Different agents were tested in different quantities: COMU (El-Faham and Albericio, 2010; Takakura et al., 2011; Chantell et al., 2012), HATU (El-Faham et al., 2010), Deoxo-Fluor Reagent (El-Faham et al., 2010), TFFH (Kangani et al., 2006), and TCFH (Carpino et al., 1996), all with a ratio of 1:1.5 and 1:3. The best yield (97%) was obtained with 1.5 equivalents of TCFH (Table 1). Other than the quantity of the different coupling reagents, all other conditions (solvent, reaction time, temperature etc.) were kept the same. Although it was not checked with chiral chromatography at this point, it became obvious following the achiral chromatography after the cysteine addition that there was no racemization, because if there had been, during either this or the previous step, we would have seen diastereomers. As the achiral chromatography after the cysteine addition was indispensable anyway, we could save the rather complicated chiral chromatography one step earlier.

**Table 1:** Coupling agents and yields.

**Coupling agent**	**Quantity**	**Yield (%)**
COMU	1.5 equiv	0
COMU	3.0 equiv	0
HATU	1.5 equiv	7
HATU	3.0 equiv	8
Deoxo-fluor reagent	1.5 equiv	48
Deoxo-fluor reagent	3.0 equiv	38
TFFH	1.5 equiv	59
TFFH	3.0 equiv	51
TCFH	1.5 equiv	97
TCFH	3.0 equiv	72

During the addition of D-cysteine (Tulla-Puche et al., 2008) to the resulting conjugate (Fmoc-Asp(OtBu)-6-amino-2-cyanobenzothiazole, **(4**), the amino acid-heterocycle conjugate was dissolved in THF and MeOH, then D-cysteine hydrochloride monohydrate was added. The resulting compound was dissolved in water, and then the cysteine was released from its salt with NaHCO_3_. During the reaction (about 25 min) the pH of the solution was kept between 7.3-7.4 by the addition of NaHCO_3_ aqueous solution, monitoring the process with a pH-meter and the addition was carried out under argon atmosphere. By this way, the desired amino acid-6-amino-D-luciferin conjugate, Fmoc-Asp(OtBu)-6-amino-D-luciferin (**5**) was obtained.

During the next step this conjugate was attached to resin (**6**). Two types of resins were tested: 2-chlorotrityl chloride and p-alkoxybenzyl alcohol (Wang resin). Loading was checked in both cases: with 2-chlorotrityl chloride resin it was 30%, while with Wang resin it was 50%, so we decided to use the latter.

Classical solid phase peptide synthesis was carried out: the peptide chain was built with Fmoc strategy. However, the *N*-terminal amino acid was always Z-protected, as this protecting group gives higher biological stability to the peptide. The obtained peptide-aLuc conjugate was removed from the resin with the mixture of TFA/water (95:5 v/v); finally, the resulting material was purified by preparative HPLC.

**Figure 5:**
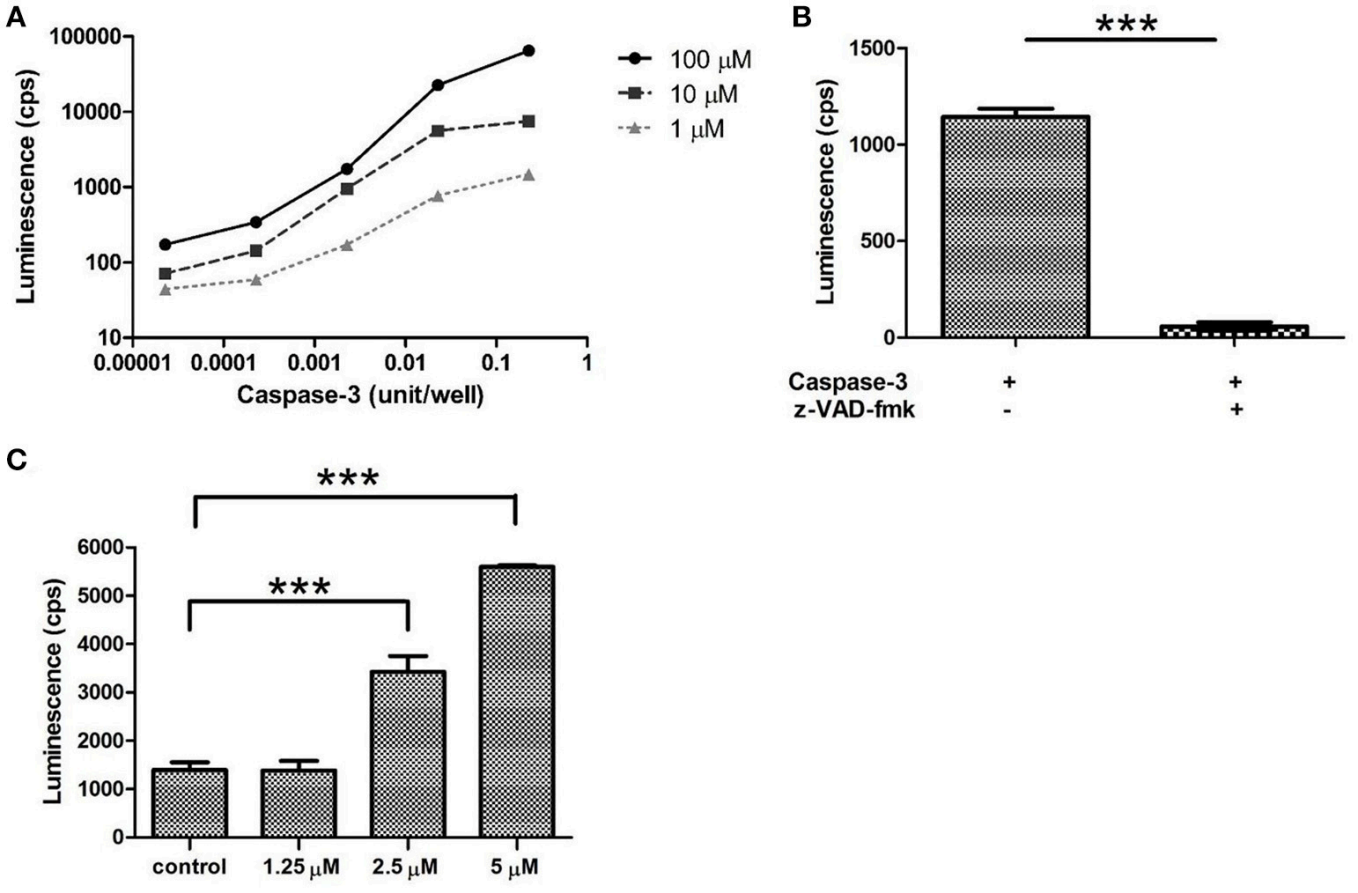
Luminescence is proportional to caspase activity and *N*-Z- DEVD-aLuc concentration. **(A)** Recombinant human caspase-3 (227 mU to 22.7 μU) and *N*-Z-DEVD-aLuc (100 to 1 μM) were titrated and assayed for 45 min. **(B)** The effect of the pan-caspase inhibitor Z-VAD-fmk was tested in equimolar ratio of *N*-Z-DEVD-aLuc at 10 μM with 2.27 mU/reaction caspase-3. **(C)** Caspase-3 and -7 activation induced by the curcumin analog C150 (5–1.25 μM) on A549 cells was detected by *N*-Z-DEVD-aLuc, control corresponds to untreated sample. The results are shown as arithmetic mean values of three samples ± SEM. Anyhow, SEM values are too small to be visible on the logarithmic scale of Figure 5A. ^***^p < 0.001.

**Figure 6:**
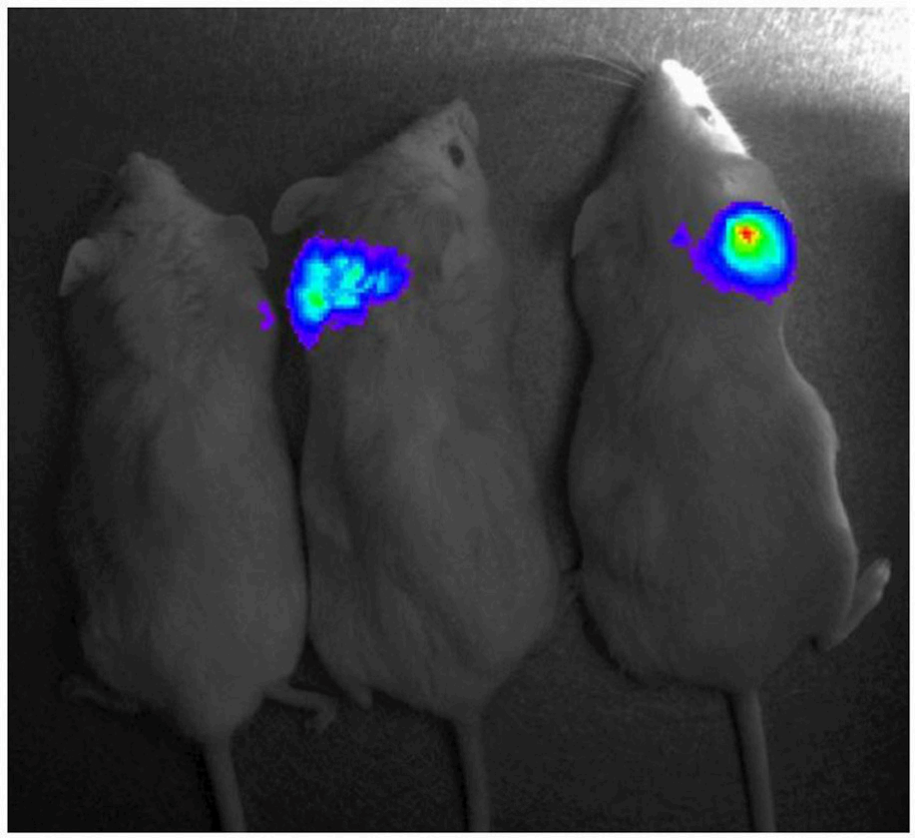
*In vivo* test of *N*-Z-DEVD-aLuc. *N*-Z-DEVD-aLuc (100 mg/kg, i.p.) was administered to all SCID mice previously inoculated with U87-Luc glioblastoma cells **(middle)**. Apoptosis was induced by Ac-915 in all mice except negative controls administered by PBS **(left)**. Aminoluciferin was used as positive control **(right)**.

The material—*N*-Z-DEVD-aLuc (**7**)—was successfully tested in a bioluminescent system. It has been published that both caspase-3 and caspase-7 digest DEVD sequence, but caspase-3 has six time higher DEVD digestion activity (Talanian et al., 1997; McStay et al., 2008) so we tested *N*-Z-DEVD-aLuc by the activity of caspase-3 *in vitro* (Figure 5) and caspase-3 *in vivo* (Figure 6). The purity of the peptide is demonstrated in Supplementary Figure 19. The biological relevance of our *N*-Z-DEVD-aLuc substrate was confirmed in a biochemical reaction using a serial dilution of recombinant caspase-3 from 227 mU/reaction to 22.7 μU/reaction. In order to verify that our *N*-Z-DEVD-aLuc is a real substrate for caspase-3, not only the enzyme but also the *N*-Z-DEVD-aLuc substrate was titrated in a concentration range from 100 to 1 μM. The recorded luminescence was linearly proportional with the growing enzyme activity and increased amount of the *N*-Z-DEVD-aLuc substrate showing maximum cps at 227 mU caspase-3 and 100 μM *N*-Z-DEVD-aLuc (Figure 5A, Supplementary Figure 20) Moreover, the luminescence signal was completely abolished by the application of equimolar pan-caspase inhibitor Z-VAD-fmk in the reaction of 2.27 mU caspase-3 and 10 μM *N*-Z-DEVD-aLuc (Ion et al., 2006; Figure 5B). We further verified the applicability of our *N*-Z-DEVD-aLuc substrate to detect cellular apoptotic cell death caused by a drug candidate molecule. The curcumin analog C150 induces caspase-3 activation (Szebeni et al., 2017; Supplementary Figure 21) and apoptosis of A549 human non-small cell lung carcinoma cells (Nagy et al., 2015; Hackler et al., 2016) and we could detect the activation of caspase-3 by *N*-Z-DEVD-aLuc via bioluminescence (Figure 5C).

To measure apoptosis directly in animals, an optical imaging experiment was performed *in vivo*, administrating *N*-Z-DEVD-aLuc to SCID mice (previously inoculated with the stably expressing luciferase cell line U87-Luc) that had been treated with chemotherapeutics previously. We used Ac-915, a lipid droplet binding thalidomide analog inducing caspase-3 activation (Supplementary Figure 22) and oxidative stress and apoptosis in different cancer cells (Nagy et al., 2013). Ac-915 enhanced the bioluminescent signal already at 6 h. Significantly fewer signals were detected from control mouse having no Ac-915 treatment, but injected with only *N*-Z-DEVD-aLuc substrate, which represents the basal level of apoptosis. However, the limitation of the widespread applicability of the luciferin conjugated peptides *in vivo* is that luciferase enzyme activity is indispensable, therefore luciferase transgenic mouse or cells should be used in these studies.

## Conclusion

A general strategy for the synthesis of *N*-peptide-6-amino-D-luciferin conjugates has been developed. The strategy is based on a newly established sequence of different transformations:

nitration → reduction → chlorine-cyanide exchange → attachment of the C-terminal amino acid of the target sequence (variable, depending on the protease to be measured) → cysteine addition → attachment to resin → solid-phase peptide synthesis (variable, depending on the protease to be measured) → cleavage from resin.

The cornerstone of *N*-peptide-6-amino-D-luciferin conjugate synthesis is the availability of 6-amino-2-cyanobenzothiazole in large quantities. Although the used transformations (nitration, NO_2_ reduction and cyanidation) are well-known in the literature, our combination of these transformations, the equipment and the solvents make it possible to prepare this material in larger quantities than the published strategies; the key of the method is the chlorine-cyano group exchange, the success of which depends on the solvent. Using this larger quantity of 6-amino-2-cyanobenzothiazole, we prepared *N*-Z-DEVD-aLuc with Fmoc solid phase peptide synthesis. The material has already been successfully used in *in vivo* optical imaging experiments.

The fact that in step 4 and 7 any amino acid can be used means that our method provides a practical and scalable way for preparation of other *N*-peptide-6-amino-D-luciferin conjugates as well, which compounds have a crucial role in the development of plate based, high-throughput viability assays.

## Author contributions

The project was conceived by LP, GT designed and coordinated the research. AK and PH chemically synthesized and analyzed the materials, GS performed *in vitro* assays. LN performed *in vivo* experiments. AK, GS, LP, and GT analyzed and compiled the data and co-wrote the manuscript. The final manuscript was read and approved by all the authors.

### Conflict of interest statement

The authors declare that the research was conducted in the absence of any commercial or financial relationships that could be construed as a potential conflict of interest. The reviewer, BL, and handling Editor declared their shared affiliation.

## Abbreviations

AcN: acetonitrile
anh: anhydrous
APCI: atmospheric pressure chemical ionization
API: atmospheric pressure ionization
CHAPS: 3-[(3-cholamidopropyl)dimethylammonio]-1-propanesulfonate
COMU: 1-cyano-2-ethoxy-2-oxoethylidenaminooxy)dimethylamino-morpholino-carbenium hexafluorophosphate
cps: counts per seconds
DCC: dicyclohexylcarbodiimide
DCM: dichloromethane
Deoxo-Fluor Reagent: bis(2-methoxyethyl)aminosulfur trifluoride
DIPEA: *N,N*-diisopropylethylamine
DMAA: *N,N*-dimethylacetamide
DMAP: 4-dimethylaminopyridine
DMEM: Dulbecco's modified eagle medium
DMEM-F12: Dulbecco's modified eagle medium nutrient mixture F-12
DMF: *N,N*-dimethylformamide
DMSO: dimethyl sulfoxide
DTT: 1,4-dithiothreitol
EDTA: disodium ethylenediaminetetraacetate dehydrate
EtOAc: ethyl acetate
FACS: fluorescence activated cell sorter
FCS: fetal calf serum
Fmoc: 9-fluorenylmethoxycarbonyl Fmoc
HATU: 1-[bis(dimethylamino)methylene]-1*H*-1,2,3-triazolo[4,5-*b*]pyridinium 3-oxid hexafluorophosphate
HEPES: 4-(2-hydroxyethyl)piperazine-1-ethanesulfonic acid
HMPA: hexamethylphosphoric acid triamide
HOBt: 1-hydroxybenzotriazole
i.p.: intraperitoneally
MeOH: methanol
mp: melting point
PBS: phosphate-buffered saline
RP-HPLC: reversed-phase high-performance liquid chromatography
SEM: standard error of the mean
SPPS: solid phase peptide synthesis
TCFH: chloro-*N,N,N*′*N*′-tetramethylformamidinium hexafluorophosphate
TFA: trifluoroacetic acid
TFFH: fluoro-*N,N,N*′,*N*′-tetramethylformamidinium hexafluorophosphate
THF: tetrahydrofuran
TLC: thin layer chromatography
Z: benzyloxycarbonyl
Z-VAD-fmk: benzyloxycarbonyl-valyl-alanyl-aspartyl-[*O*-methyl]- fluoromethylketone.
