# Supplementary material for: Synthesis of N-peptide-6-amino-D-luciferin Conjugates
Source: Front Chem. 2018 Apr 19;6:120. doi: 10.3389/fchem.2018.00120 (PMC5917020; doi:10.3389/fchem.2018.00120)
Supplement: Supplementary file 1 [file Table1.PDF]

Supplementary Material

Synthesis of *N*-peptide-6-amino-D-luciferin Conjugates

Anita. K. Kovács[1, 2*], Péter Hegyes[2], Gábor. J. Szebeni[2, 3], Lajos I. Nagy[2], László G. Puskás[2, 3], Gábor K. Tóth[1*]

[1] Department of Medical Chemistry, University of Szeged, Szeged, Hungary

[2]Avidin Ltd, Szeged, Hungary

[3]Department of Genetics, Biological Research Center, Hungarian Academy of Sciences, Szeged, Hungary

***Correspondence:**
Anita K. Kovács
kovacs.anita@med.u-szeged.hu
Gábor K. Tóth
toth.gabor@med.u-szeged.hu

1. Supplementary Tables **2-10.**

2. Supplementary Figures **11-35.**

3. Supplementary Materials and Methods **36.**

Supplementary Table 1.1 Methods for the synthesis of 6-amino-2-cyanobenzothiazole

Author	Starting material	Transformation	Yield (%)	Overall yield	Reagent	Solvent	Reaction time	Temperature (°C)	Disadvantages	Comments
Takakura et al. 2011	2-chlorobenzothiazole	nitration	49	**16**	H_2SO_4, KNO_3	H_2SO_4	1h 30m	0-15	-	
	2-chloro-6-nitrobenzothiazole	reduction	61		$SnCl_2$, HCl, NaOH	EtOH, H_2O	?	120	A significant amount of by-product (2-hydroxy-6-aminobenzothiazole and 6-aminobenzothiazole) formed. The high temperature cannot be understood.	
	6-amino-2-chlorobenzothiazole	cyanidation	54		KCN	DMSO	overnight	135	DMSO is not the best solvent for this step, as with distillation it cannot be regained from the aqueous solution.	
Gryshuk et al. 1 2013	6-nitrobenzothiazole	acylation	61	**8**	H_2SO_4, H_2O_2, $FeSO_4$, $NaHCO_3$	H_2O	more than 30m	rt, 0	The step itself is good, but the starting material is ill-chosen.	The starting material is not optimal, consequently the method is too complicated, the desired end-product is reached in four steps, instead of the others' three.
	6-nitrobenzothiazole-2-carboxylate	amidation	80		NH_3	MeOH	20m	rt	-	
	6-nitrobenzothiazole-2-carboxamide	cyanidation	24		$POCl_3$, H_2O, pyridine	EtOAc	2h 20m	0, rt	-	
	2-cyano-6-nitrobenzothiazole	reduction	65		$SnCl_2$, $NaHCO_3$	EtOH, H_2O	2h	rt, 60	If the cyano group is present during the NO_2 reduction, there is a high risk of its reduction into an aminomethyl group.	

Supplementary Table 1.2 Methods for the synthesis of 6-amino-2-cyanobenzothiazole

Author	Starting material	Transformation	Yield (%)	Overall yield	Reagent	Solvent	Reaction time	Temperature (°C)	Disadvantages	Comments
Gryshuk et al. 2 2013	ethyl benzothiazole-2-carboxylate	nitration	10	**5**	H_2SO_4, HNO_3	H_2O	more than 1h	0-15	The ill-chosen starting material can be nitrated with very low yield, also, there is a high risk of hydrolysis under the aqueous-acidic conditions.	The desired end-product is reached in four steps, instead of the others' three.
	ethyl-6-nitrobenzothiazole-2-carboxylate	amidation	80		NH_3	MeOH	20m	rt	-	
	6-nitrobenzothiazole-2-carboxamide	cyanidation	24		$POCl_3$, H_2O, pyridine	EtOAc	2h 20m	0, rt	-	
	2-cyano-6-nitrobenzothiazole	reduction	65		$SnCl_2$, $NaHCO_3$	EtOH, H_2O	2h	rt, 60	If the cyano group is present during the NO_2 reduction, there is a high risk of its reduction into an aminomethyl group.	
Gryshuk et al. 3 2013	ethyl benzothiazole-2-carboxylate	amidation	?	**(<5)**	NH_3	MeOH	15-30m	rt	-	In step 1 there is 100% conversion rate, but due to the lack of purification, no known yield.
	benzothiazole-2-carboxamide	nitration	30		H_2SO_4, HNO_3	H_2O	overnight	0-15	There is a risk of hydrolysis of the amide under the aqueous-acidic conditions.	
	6-nitrobenzothiazole-2-carboxamide	cyanidation	24		$POCl_3$, H_2O, pyridine	EtOAc	2h 20m	0, rt	-	
	2-cyano-6-nitrobenzothiazole	reduction	65		$SnCl_2$, $NaHCO_3$	EtOH, H_2O	2h	rt, 60	If the cyano group is present during the NO_2 reduction, there is a high risk of its reduction into an aminomethyl group.	

Supplementary Table 1.3 Methods for the synthesis of 6-amino-2-cyanobenzothiazole

Author	Starting material	Transformation	Yield (%)	Overall yield	Reagent	Solvent	Reaction time	Temperature (°C)	Disadvantages	Comments
Gryshuk et al. 4 2013	ethyl benzothiazole-2-carboxylate	amidation	?	**(<62)**	NH_3	MeOH	15-30m	rt	-	In step 1, 100% conversion, but due to the lack of purification, no known yield, In step 3, yield was not determined, therefore the overall yield cannot be determined.
	benzothiazole-2-carboxamide	cyanidation	95		$POCl_3$, H_2O, pyridine	EtOAc	2h 20m	0, rt	-	
	2-cyanobenzothiazole	nitration	?		H_2SO_4, HNO_3	H_2O	6h	0	-	
	2-cyano-6-nitrobenzothiazole	reduction	65		$SnCl_2$, $NaHCO_3$	EtOH, H_2O	2h	rt, 60	If the cyano group is present during the NO_2 reduction, there is a high risk of its reduction into an aminomethyl group.	
McCutcheon et al. 1 2015	4-nitroaniline	thiocyanidation	80	**56**	pyridine, sodium thiosulphate, 4,5-dichloro-1,2,3-dithiazol-1-ium chloride	H_2O, acetonitrile, THF	1h 30m	rt	Complicated method, fewer reagents would be more reasonable. 4,5-dichloro-1,2,3-dithiazol-1-ium chloride is very expensive.	Extremely complicated route.
	(4-nitrophenyl)-carbonocyanidothioic amide	cyanidation	74		CuI, Bu_4N^+ •Br-, Catalyst: $PdCl_2$	DMF, DMSO	4h	130	Extremely complicated method, special catalysts are required.	
	6-nitro-2-cyanobenzothiazole	reduction	95		Zn, NH_4Cl	MeOH	35m	rt	If the cyano group is present during the NO_2 reduction, there is a high risk of its reduction into an aminomethyl group.	

Supplementary Table 1.4 Methods for the synthesis of 6-amino-2-cyanobenzothiazole

Author	Starting material	Transformation	Yield (%)	Overall yield	Reagent	Solvent	Reaction time	Temperature (°C)	Disadvantages	Comments
McCutcheon et al. 2 2015	6-nitrobenzothiazole	decomposition of the thiazole ring	62	**37**	N_2H_4	EtOH, DCM	12h	rt, reflux	-	
	2-amino-5-nitrobenzenethiol	cyanidation	62		4,5-dichloro-1,2,3-dithiazol-1-ium chloride	DCM	12 h	reflux	4,5-dichloro-1,2,3-dithiazol-1-ium chloride is very expensive.	
	6-nitro-2-cyanobenzothiazole	reduction	95		NH_4Cl, Zn	MeOH	35m	rt	If the cyano group is present during the NO_2 reduction, there is a high risk of its reduction into an aminomethyl group.	
Hsu et al. 2016	2-chlorobenzothiazole	nitration	67	**27**	H_2SO_4, KNO_3	H_2SO_4	4h	0-15	-	Too much by-product and waste.
	2-chloro-6-nitrobenzothiazole	reduction	88		Fe, $FeCl_3$	EtOH, acetic acid	24h	80	The resulting by-product (Fe(III)-acetate) is difficult to separate from the desired product.	
	6-amino-2-chlorobenzothiazole	cyanidation	45		KCN	DMSO	24h	120	DMSO is not the best solvent for this step, as with distillation it cannot be regained from the aqueous solution.	
Hauser et al. 2016	2-chlorobenzothiazole	nitration	82	**44**	H_2SO_4, KNO_3	H_2SO_4	18h	0-15	-	Too much by-product.
	2-chloro-6-nitrobenzothiazole	cyanidation	75		NaCN, Dabco catalyst, $FeCl_3$	H_2O, acetonitrile	24h	rt	Two byproducts (2-carbethoxy-6-nitrobenzothiazole and 2-ethoxy-nitrobenzothiazole) formed.	
	2-cyano-6-nitrobenzothiazole	reduction	71		Fe	acetic acid	24h	rt	If the cyano group is present during the NO_2 reduction, also, there is a high risk of its reduction into an aminomethyl group. The resulting by-product (Fe(III)-acetate) is difficult to separate from the desired product.	

Supplementary Table 2 Comparison of starting materials

Author	Starting material	Transformation	Yield (%)	Reagent	Solvent	Reaction time	Temperature (°C)	Disadvantages	Comments
Takakura et al. 2011	2-chlorobenzothiazole	nitration	49	H_2SO_4, KNO_3	H_2SO_4	1h 30m	0-15	–	
Gryshuk et al. 1 2013	6-nitrobenzothiazole	acylation	61	H_2SO_4, H_2O_2, $FeSO_4$, $NaHCO_3$	H_2O	more than 30m	rt, 0	The step itself is good, but the starting material is ill-chosen, which makes the following step uneconomical.	.
Gryshuk et al. 2 2013	ethyl benzothiazole-2-carboxylate	nitration	10	H_2SO_4, HNO_3	H_2O	more than 1h	0-15	The ill-chosen starting material can be nitrated with very low yield, also, there is a high risk of hydrolysis under the aqueous-acidic conditions.	
Gryshuk et al. 3 2013	ethyl benzothiazole-2-carboxylate	amidation	?	NH_3	MeOH	15-30m	rt	–	100% conversion, but due to the lack of purification, yield was not determined
Gryshuk et al. 4 2013	ethyl benzothiazole-2-carboxylate	amidation	?	NH_3	MeOH	15-30m	rt	–	100% conversion, but due to the lack of purification, yield was not determined
McCutcheon et al. 1 2015	4-Nitroaniline	thiocyanidation	80	pyridine, sodium thiosulphate, 4,5-dichloro-1,2,3-dithiazol-1-ium chloride	H_2O, acetonitrile, THF	1h 30m	rt	Complicated method, fewer reagents would be more reasonable. 4,5-dichloro-1,2,3-dithiazol-1-ium chloride is very expensive.	
McCutcheon et al. 2 2015	6-nitrobenzothiazole	decomposition of the thiazole ring	62	N_2H_4	EtOH, DCM	12h	rt, reflux	–	
Hsu et al. 2016	2-chlorobenzothiazole	nitration	67	H_2SO_4, KNO_3	H_2SO_4	4h	0-15	–	
Hauser et al. 2016	2-chlorobenzothiazole	nitration	82	H_2SO_4, KNO_3	H_2SO_4	18h	0-15	–	

Supplementary Table 3 Comparison of solvents during chlorine-cyanide exchange

Author	Starting material	Transformation	Yield (%)	Reagent	Solvent	Reaction time	Temperature (°C)	Disadvantages
Takakura et al. 2011	6-amino-2-chlorobenzothiazole	cyanidation	54	KCN	DMSO	overnight	135	DMSO is not the best solvent for this step, as with distillation it cannot be regained from the aqueous solution.
Hsu et al. 2016	6-amino-2-chlorobenzothiazole	cyanidation	45	KCN	DMSO	24h	120	DMSO is not the best solvent for this step, as with distillation it cannot be regained from the aqueous solution.
Hauser et al. 2016	2-chloro-6-nitrobenzothiazole	cyanidation	75	NaCN, Dabco catalyst, $FeCl_3$	H_2O, acetonitrile	24h	rt	Two byproducts (2-carbethoxy-6-nitrobenzothiazole and 2-ethoxy-nitrobenzothiazole) formed.

Supplemenary Table 4 Comparison of the optimal and the non-optimal order of reduction and cyanidation

Author	Starting material	Transformation	Yield (%)	Reagent	Solvent	Reaction time	Temperature (°C)	Disadvantages
Takakura et al. 2011	2-chloro-6-nitrobenzothiazole	reduction	61	$SnCl_2$, HCl, NaOH	EtOH, H_2O	?	120	-
	6-amino-2-chlorobenzothiazole	cyanidation	54	KCN	DMSO	overnight	135	
Gryshuk et al. 1, 2 and 3 2013	6-nitrobenzothiazole-2-carboxamide	cyanidation	24	$POCl_3$, H_2O, pyridine	EtOAc	2h 20m	0, rt	If the cyano group is present during the NO_2 reduction, there is a high risk of its reduction into an aminomethyl group.
	2-cyano-6-nitrobenzothiazole	reduction	65	$SnCl_2$, $NaHCO_3$	EtOH, H_2O	2h	rt, 60	
Gryshuk et al. 4 2013	benzothiazole-2-carboxamide	cyanidation	95	$POCl_3$, H_2O, pyridine	EtOAc	2h 20m	0, rt	If the cyano group is present during the NO_2 reduction, there is a high risk of its reduction into an aminomethyl group.
	2-cyanobenzothiazole	nitration	?	H_2SO_4, HNO_3	H_2O	6h	0	
	2-cyano-6-nitrobenzothiazole	reduction	65	$SnCl_2$, $NaHCO_3$	EtOH, H_2O	2h	rt, 60	
McCutcheon et al. 1 2015	(4-nitrophenyl)-carbonocyanidothioic amide	cyanidation	74	CuI, Bu_4N^+ •Br-, Catalyst: $PdCl_2$	DMF, DMSO	4 h	130	If the cyano group is present during the NO_2 reduction, there is a high risk of its reduction into an aminomethyl group.
	6-nitro-2-cyanobenzothiazole	reduction	95	Zn, NH_4Cl	MeOH	35m	rt	
McCutcheon et al 2. 2015	2-amino-5-nitrobenzenethiol	cyanidation	62	4,5-dichloro-1,2,3-dithiazol-1-ium chloride	DCM	12 h	reflux	If the cyano group is present during the NO_2 reduction, there is a high risk of its reduction into an aminomethyl group.
	6-nitro-2-cyanobenzothiazole	reduction	95	NH_4Cl, Zn	MeOH	35m	rt	
Hauser et al. 2016	2-chloro-6-nitrobenzothiazole	cyanidation	75	NaCN, Dabco catalyst, $FeCl_3$	H_2O, acetonitrile	24h	rt	If the cyano group is present during the NO_2 reduction, also, there is a high risk of its reduction into an aminomethyl group. The resulting by-product (Fe(III)-acetate) is difficult to separate from the desired product..
	2-cyano-6-nitrobenzothiazole	reduction	71	Fe	acetic acid	24h	rt	

Supplementary Table 5 Synthesis method of *N*-peptide-6-amino-D-luciferin conjugates by Gryshuk et al. (2011)

Author	Starting material	Transformation	Yield (%)	Overall yield	Reagent	Solvent	Reaction time	Temperature (°C)	Disadvantages	Comments
Gryshuk et al. 2011	6-amino-2-cyanobenzothiazole	amide bond formation (acylation)	?	?	Boc-Tyr(tBu)-OH, *N*-methyl morpholine, isobutyl chloroformate	anh THF	2.5h, overnight	0, rt	The mixed anhydride method is not optimal, because it is not economical. Yield was not determined.	Besides the risk of racemization, it is hard to evaluate the route, as yield was not determined throughout the route.
	Boc-Tyr(tBu)-6-amino-2-cyanobenzothiazole	Boc deprotection	?		20% TFA	DCM	2h	rt	After the Boc-deprotection the mixture is purified but yield is not determined.	
	H-Tyr-6-amino-2-cyanobenzothiazole	amide bond formation (acylation)	?		protected peptide sequence, *N*-methyl morpholine, isobutyl chloroformate	anh THF, anh DMF	2.5h, 72h	0, rt	The mixed anhydride method is not optimal, because it is not economical. Yield was not determined.	
	protected peptide-Tyr-6-amino-2-cyanobenzothiazole	deprotection of side chains of the peptide portion	?		50% TFA	anh DCM	3h	rt	Neither conversion, nor yield is determined.	
	deprotected peptide-Tyr-6-amino-2-cyanobenzothiazole	thiazoline ring formation (Cys addition)	?		H-D-Cys-OH, K_2CO_3	anh THF	2h	rt	The purification of the deprotected peptide-6-amino-2-cyanobenzothiazole is unnecessary before the Cys addition. The reaction is at pH 8, there is a high risk of the racemization of Cys..	

Supplementary Table 6 Synthesis method of *N*-peptide-6-amino-D-luciferin conjugates by Kovács et al. (2018)

Author	Starting material	Transformation	Yield (%)	Overall yield	Reagent	Solvent	Reaction time	Temperature (°C)	Disadvantages	Comments
Kovács et al 2018. (submitted)	2-chlorobenzothiazole	nitration	83	57	H_2SO_4, KNO_3	H_2SO_4	5h	0-15	-	-
	2-chloro-6-nitrobenzothiazole (1)	reduction	88		NH_4Cl, H_2O, Fe powder	EtOAc	8h	reflux	-	Using Soxhlet extractor, the EtOAc can be fully regained.
	6-amino-2-chlorobenzothiazole (2)	cyanidation	78		KCN	DMAA	12h	110	The reagent is toxic, but was reacted with KH_2PO_4 in order to get non-toxic KOCN.	The use of DMAA instead of other solvents results in significantly better yield during cyanidation.
	6-amino-2-cyanobenzothiazole (3)	amide bond formation (acylation, attachment of the C-terminal amino acid of the target sequence)	73	28	Fmoc-Asp(OtBu)-OH, TCFH, DIPEA	dry DCM	overnight	rt	-	TCFH is very effective when forming amide bond.
	Fmoc-Asp(OtBu)-6-amino-2-cyanobenzothiazole (4)	thiazoline ring formation (Cys addition)	81		D-cysteine·HCl·H2O, 5% (m/m) $NaHCO_3$	MeOH,THF, H_2O	2h	rt	-	Keeping pH under 7.4, racemization can be prevented.
	Fmoc-Asp(OtBu)-6-amino-D-luciferin (5)	attachment to resin	47.8		p-alkoxybenzyl alcohol resin, DCC, HOBt, DMAP	dry DCM, MeOH	5h 30m	rt	-	p-alkoxybenzyl alcohol resin gives the highest load.
	Fmoc-Asp(OtBu)-6-amino-D-luciferin-resin (6)	solid-phase peptide synthesis	(nonapplicable)	-	1; 20% (v/v) piperidine<br>2; Fmoc-AA-OH (N-terminal amino acid is Z-AA-OH), DCC, HOBt	1; DMF, MeOH<br>2; DMF, MeOH	1; 20m<br>2; 2 h	1; rt<br>2; rt	-	**In theory any peptide sequence of any length can be built at this step. The *N*-terminal amino acid must be Z-protected, as this protecting group gives higher biological stability to the peptide.**
	N-Z-DEVD-6-amino-D-luciferin-resin	cleavage from resin	(nonapplicable)	-	TFA	H_2O	2h	rt	-	-

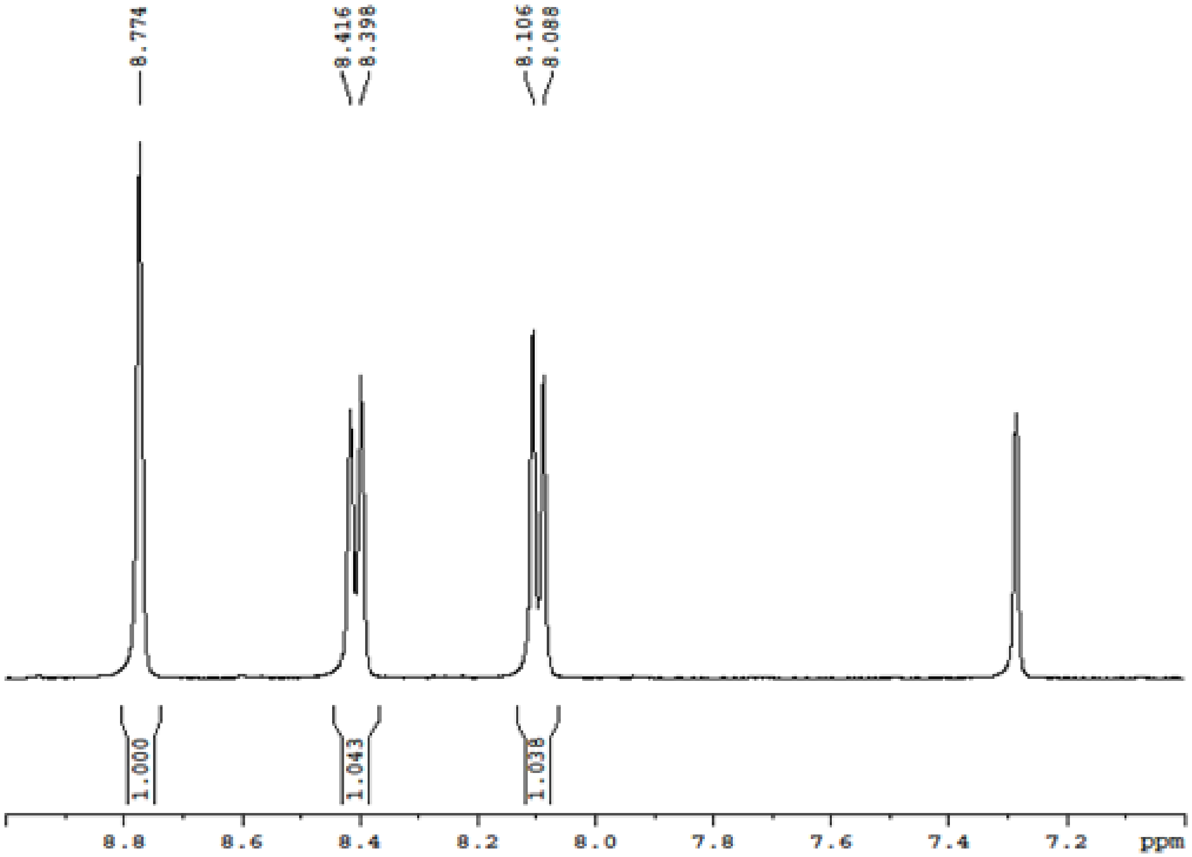

Supplementary Figure 1 ^{1}H NMR spectrum of 2-chloro-6-nitrobenzothiazole (**1**)

11

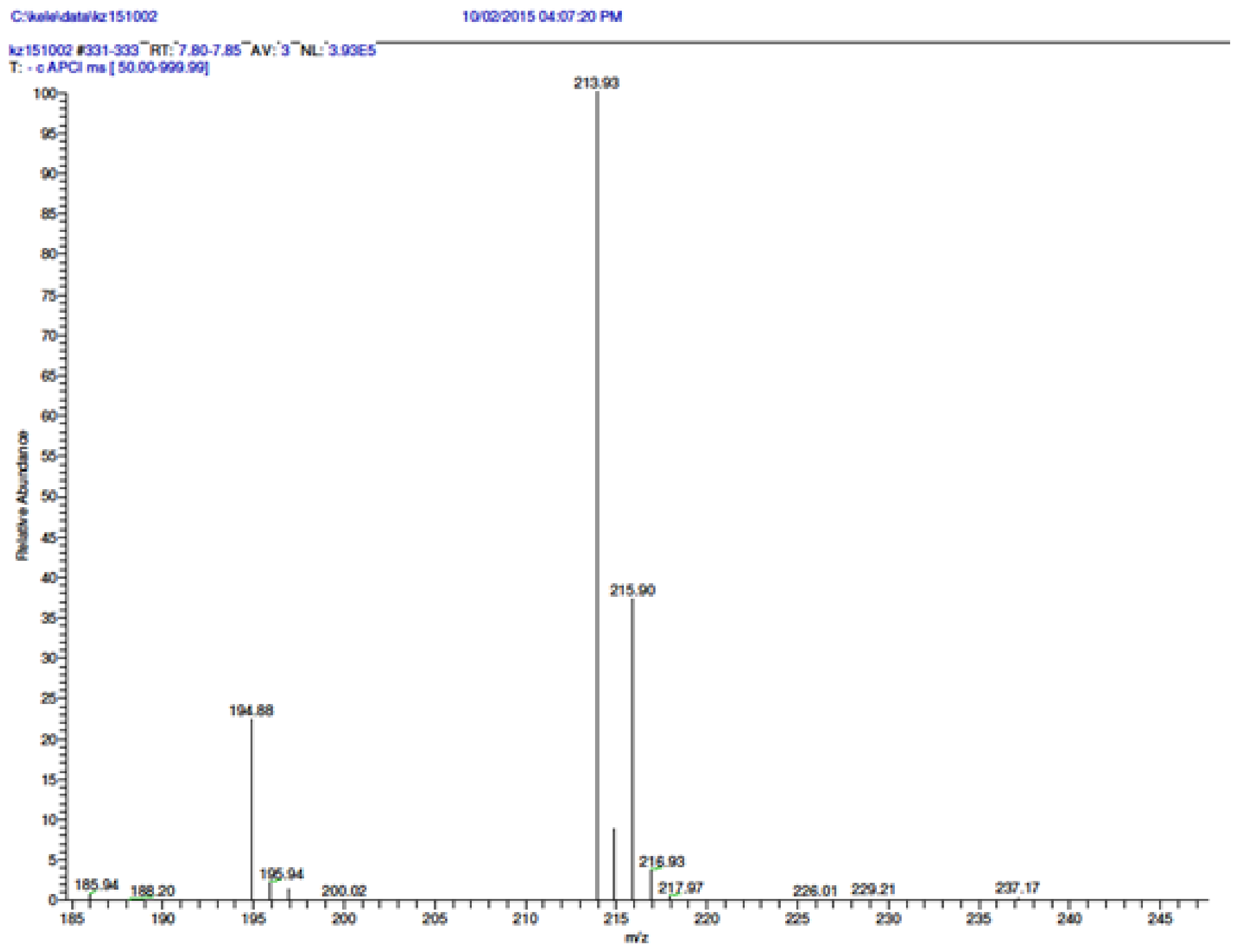

Supplementary Figure 2 Mass spectrum (TSQ) of 2-chloro-6-nitrobenzothiazole (**1**) 213.93= $[M-H]^-$

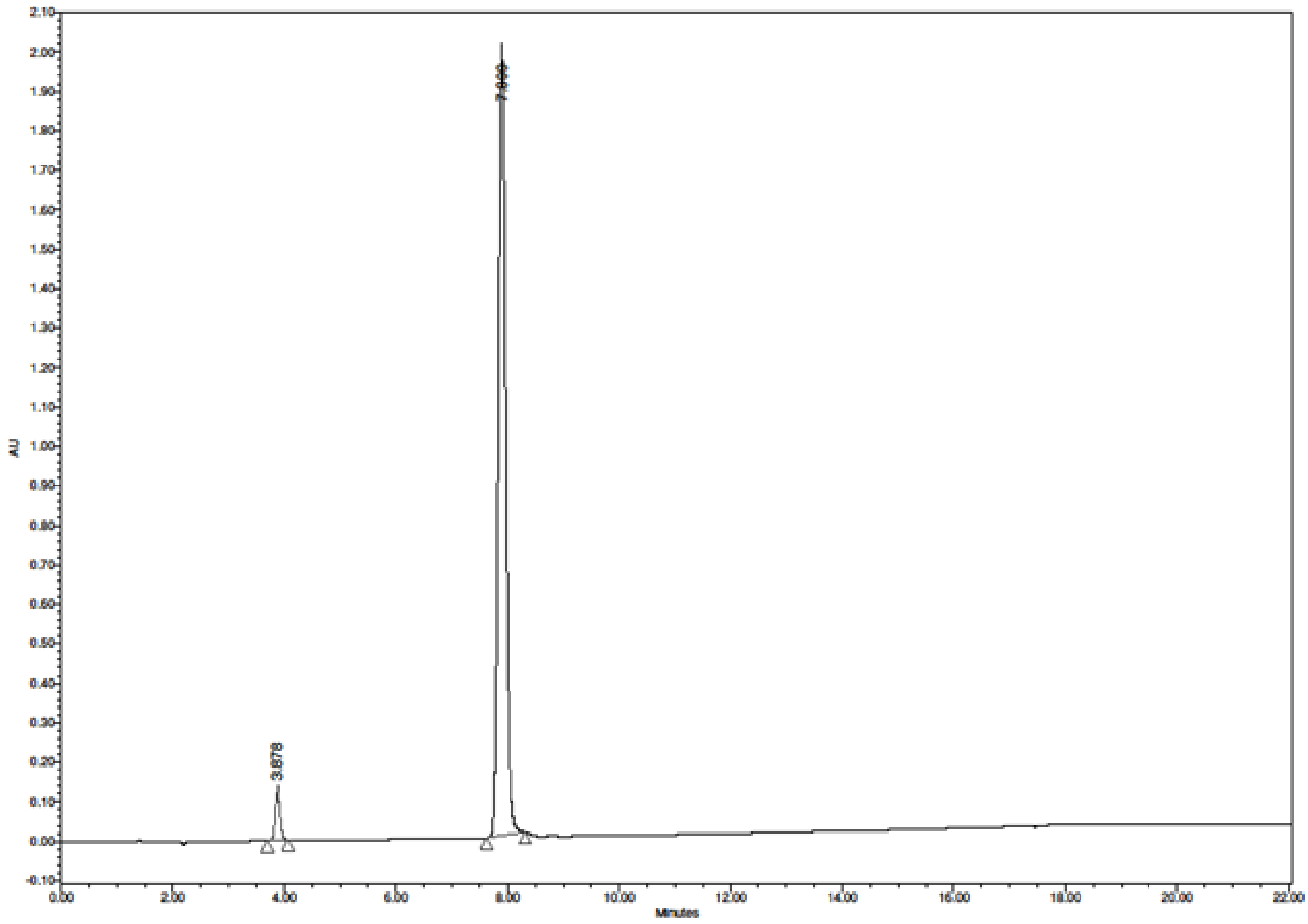

Supplementary Figure 3 RP-HPLC profile of the crude 2-chloro-6-nitrobenzothiazole (**1**) 70-100% B in 15 min + 100% B in 5 min, t_{R1}= 3.878 min: 2-chloro-5-nitrobenzothiazole t_{R2}= 7.899 min: 2-chloro-6-nitrobenzothiazole (**1**)

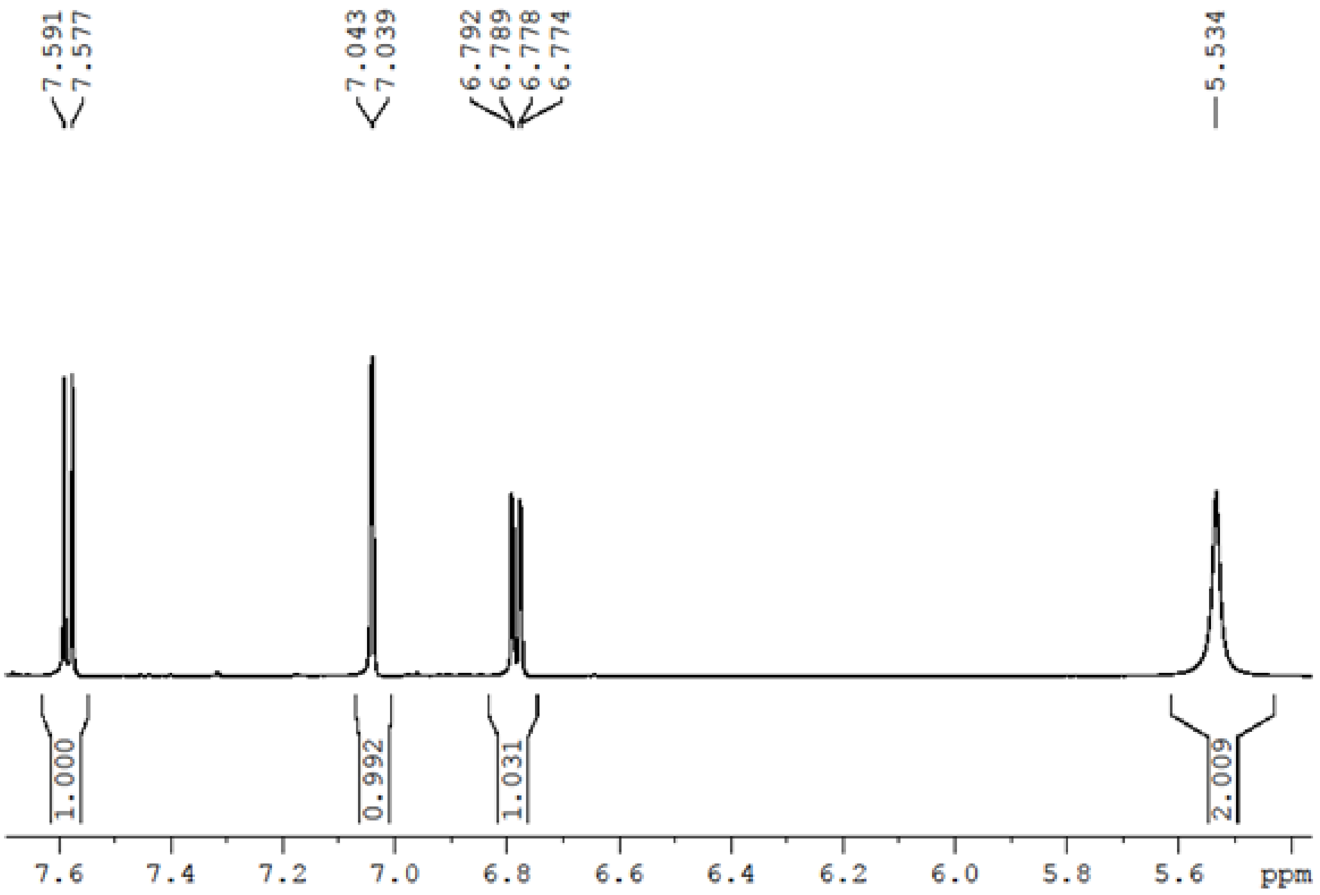

Supplementary Figure 4 ^{1}H NMR spectrum of 6-amino-2-chlorobenzothiazole (**2**)

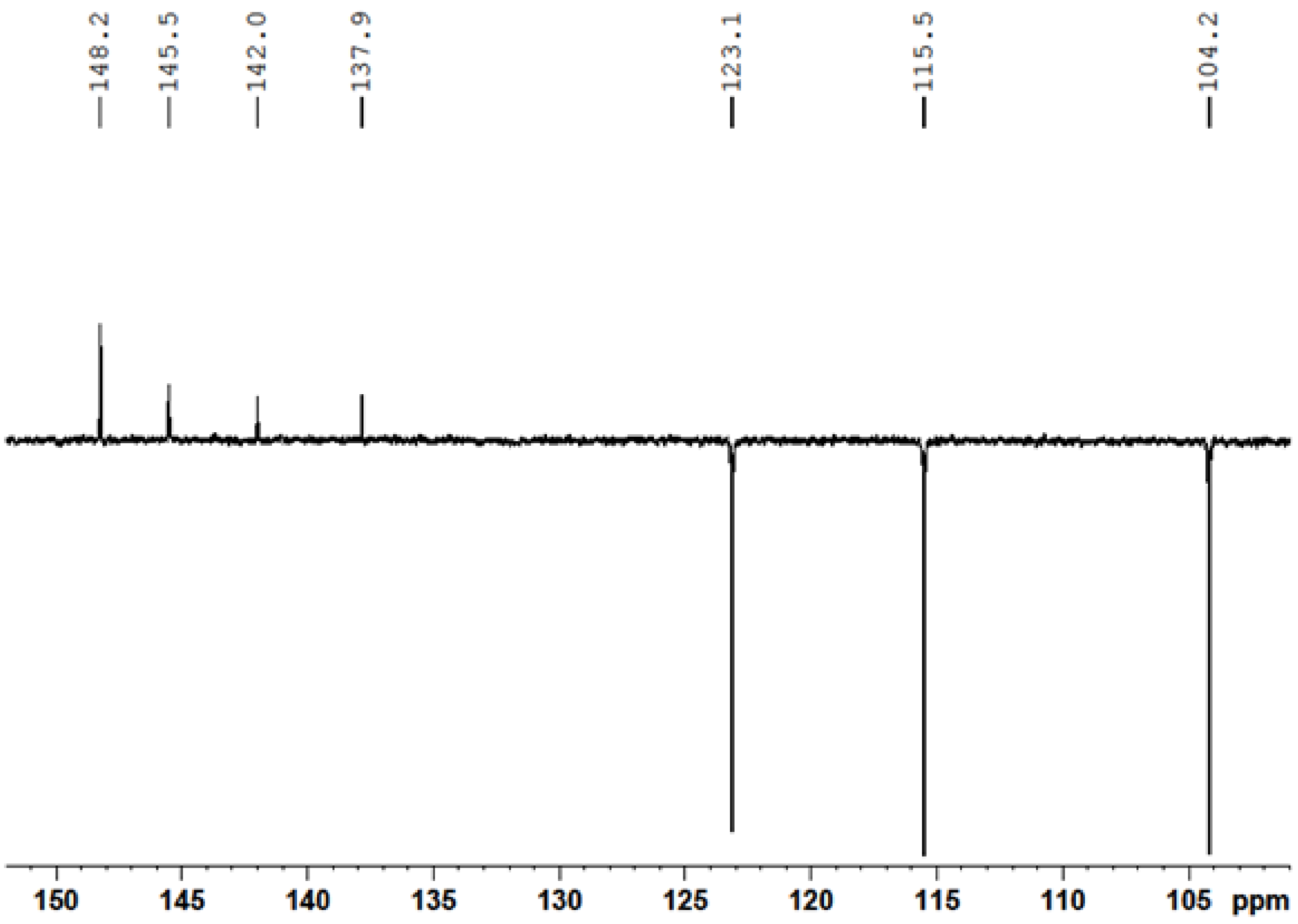

Supplementary Figure 5 ^{13}C NMR spectrum of 6-amino-2-chlorobenzothiazole (**2**)

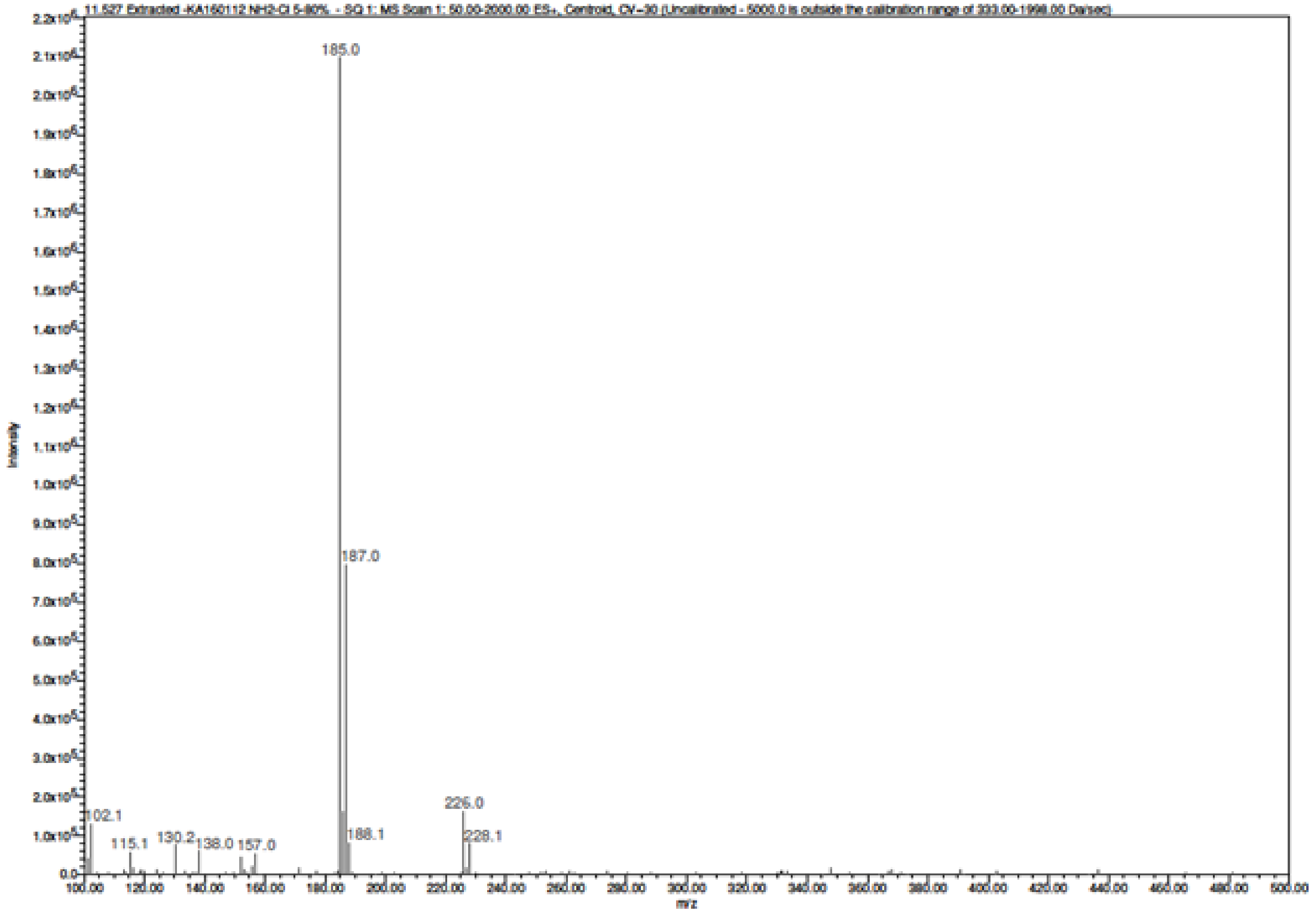

Supplementary Figure 6 Mass spectrum (ESI) of 6-amino-2-chlorobenzothiazole (**2**) 185.0= $[M+H]^+$

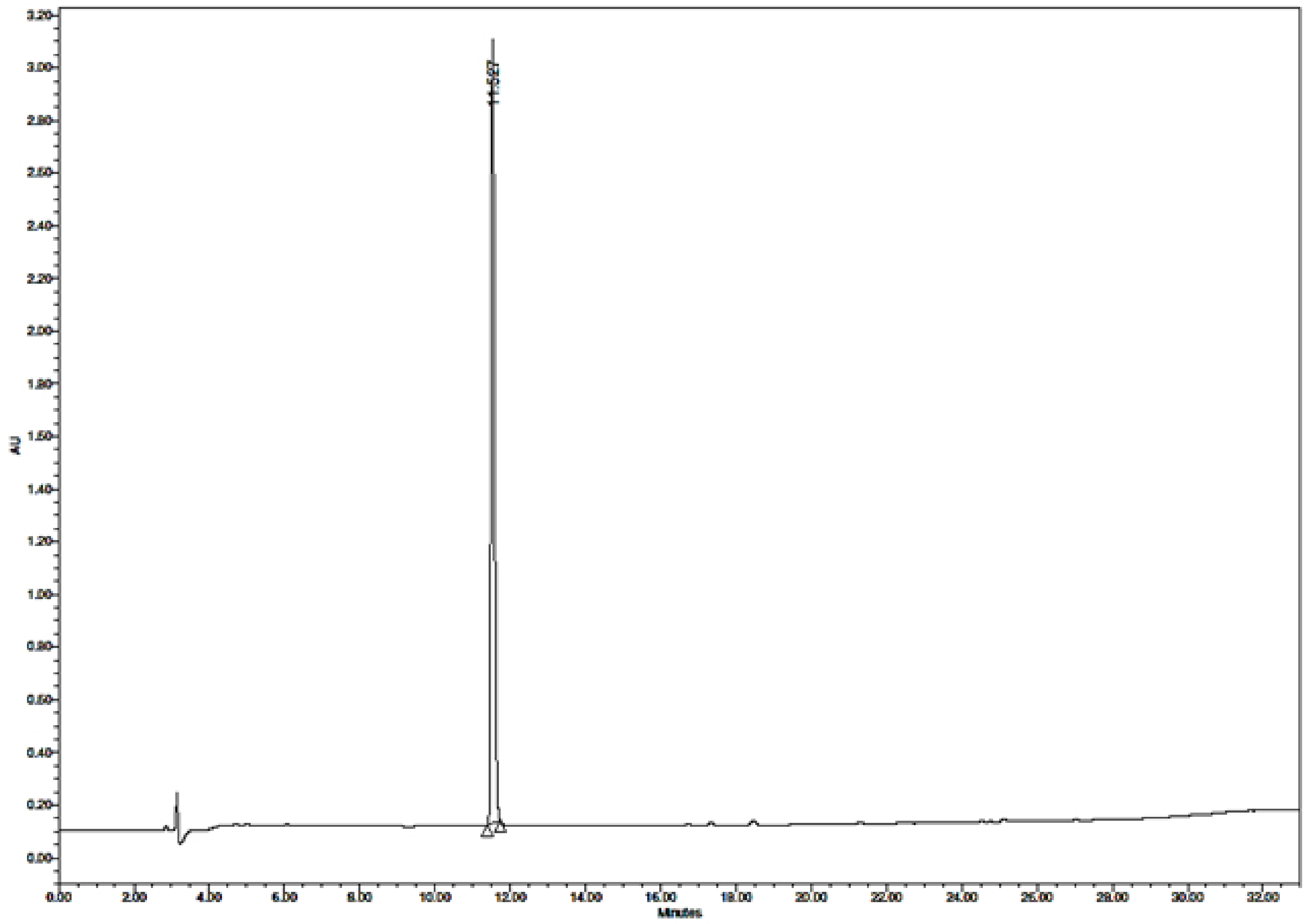

Supplementary Figure 7 RP-HPLC profile of 6-amino-2-chlorobenzothiazole (**2**)
5-80% B in 25 min + 3 min up to 100% B + 100% B in 5 min, t_R= 11.527 min

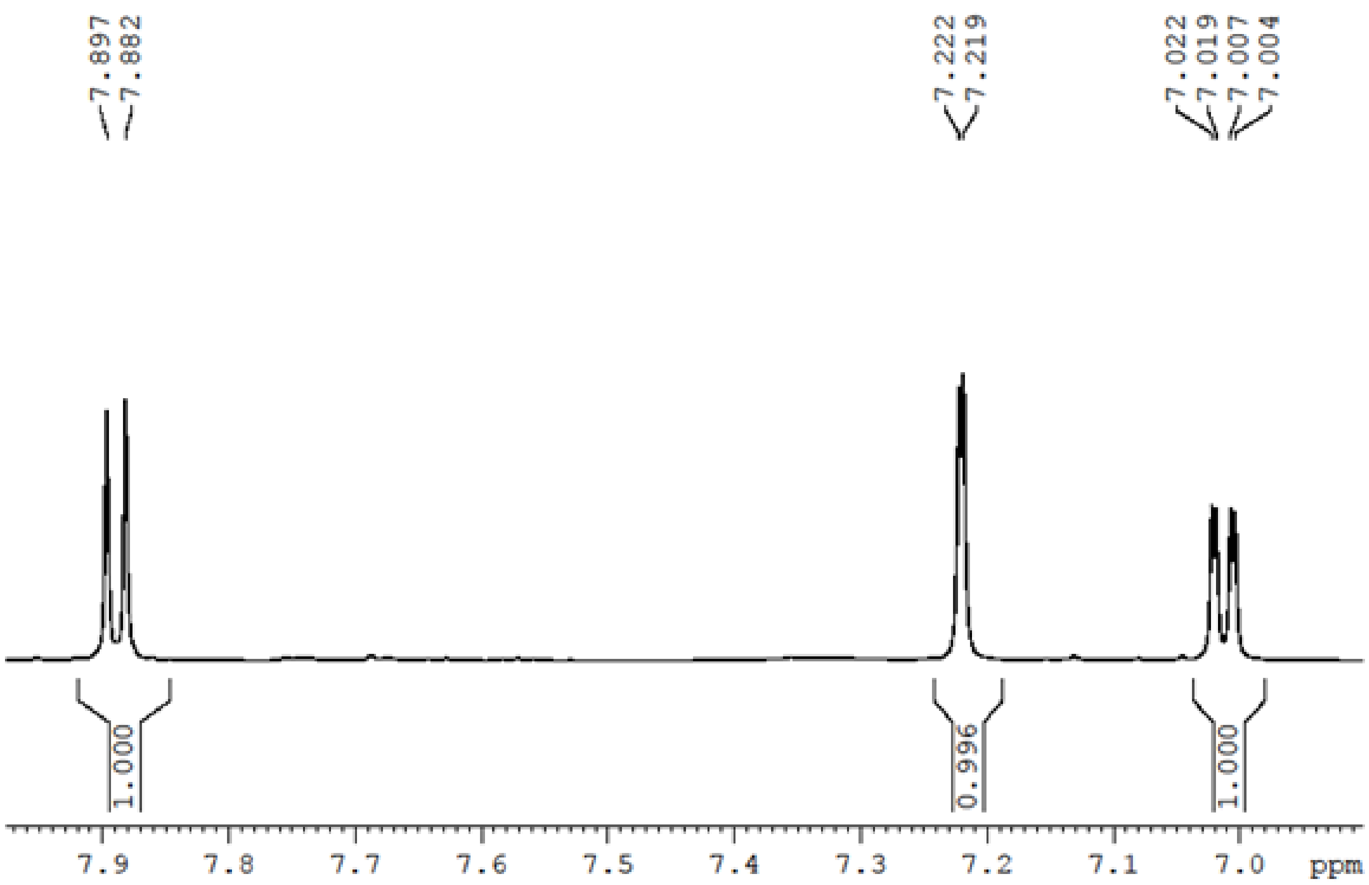

Supplementary Figure 8 ^{1}H NMR spectrum of 6-amino-2-cyanobenzothiazole (**3**)

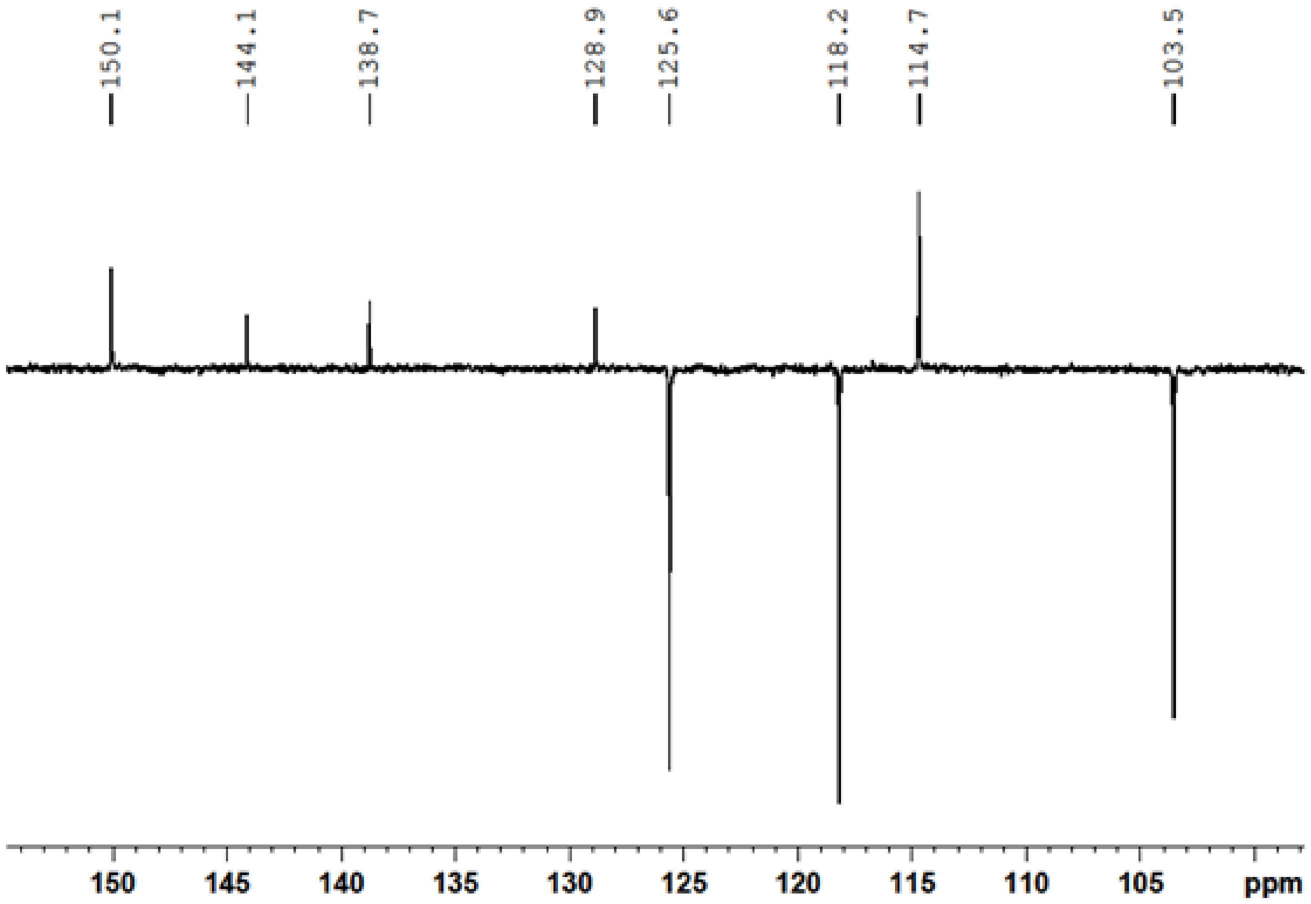

Supplementary Figure 9 ^{13}C NMR spectrum of 6-amino-2-cyanobenzothiazole (**3**)

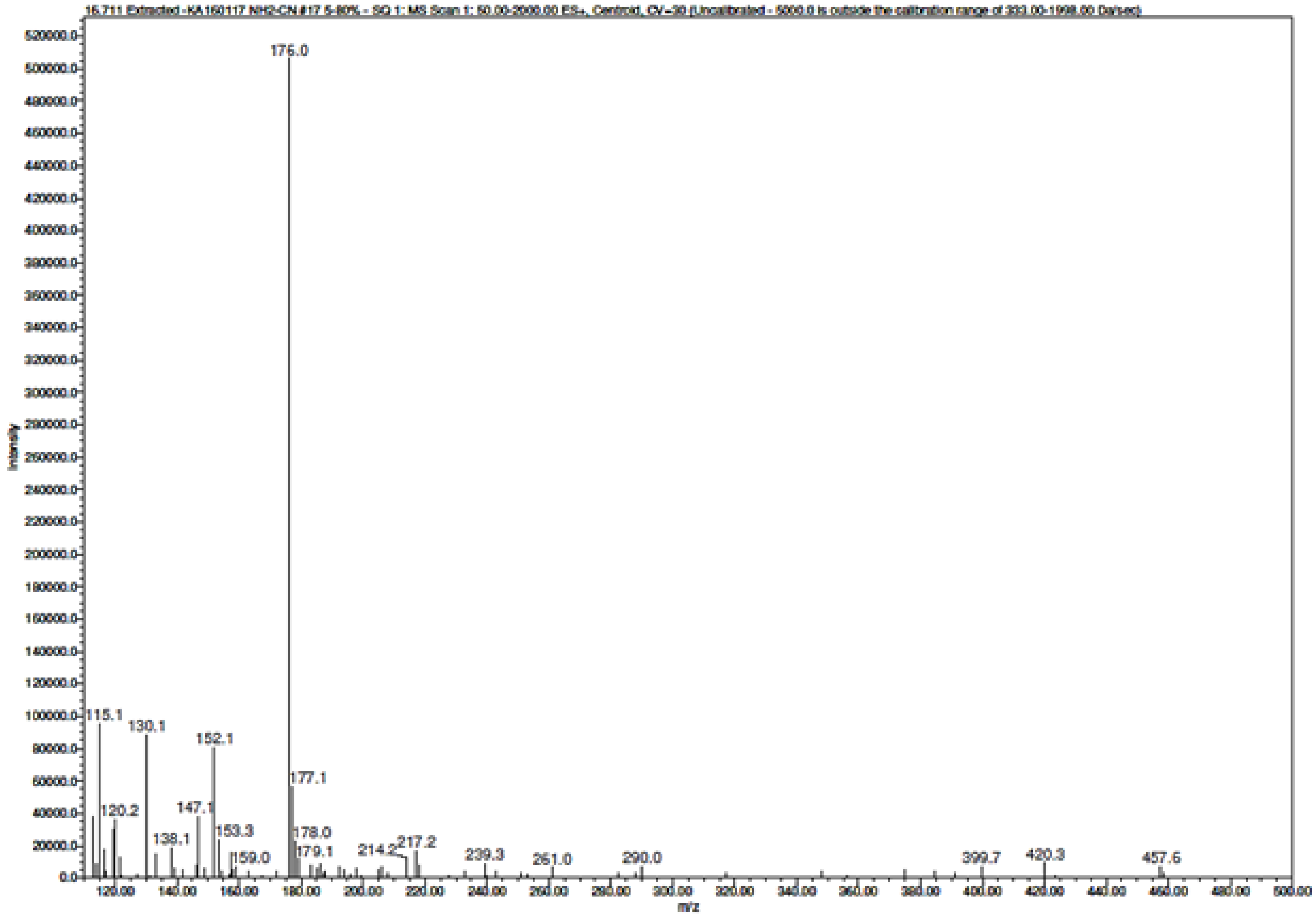

Supplementary Figure 10 Mass spectrum (ESI) of 6-amino-2-cyanobenzothiazole (**3**) 176.0= $[M+H]^+$

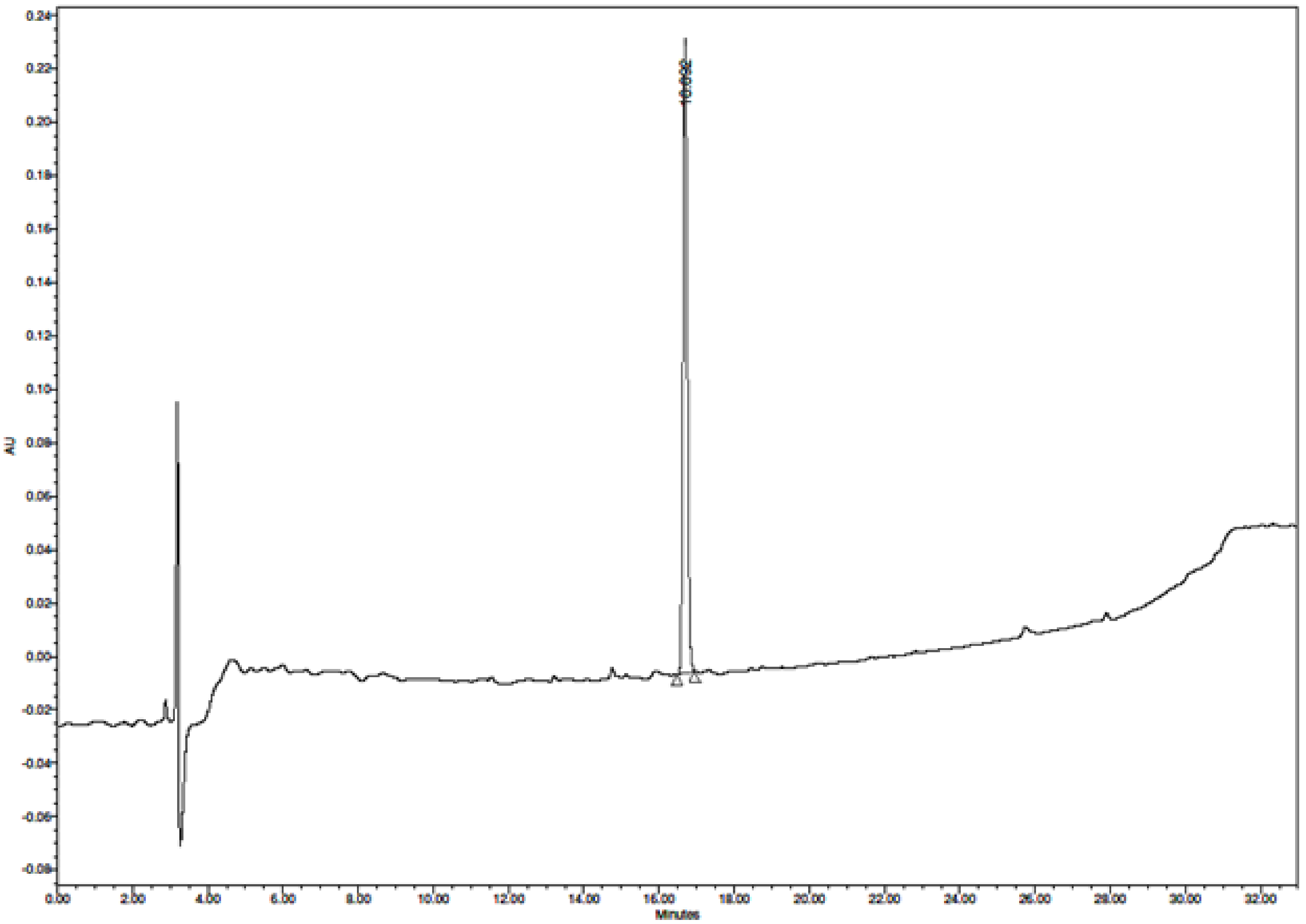

Supplementary Figure 11 RP-HPLC profile of 6-amino-2-cyanobenzothiazole(**3**)
5-80% B in 25 min + 3 min up to 100% B + 5 in 100% B, t_R= 16.692 min

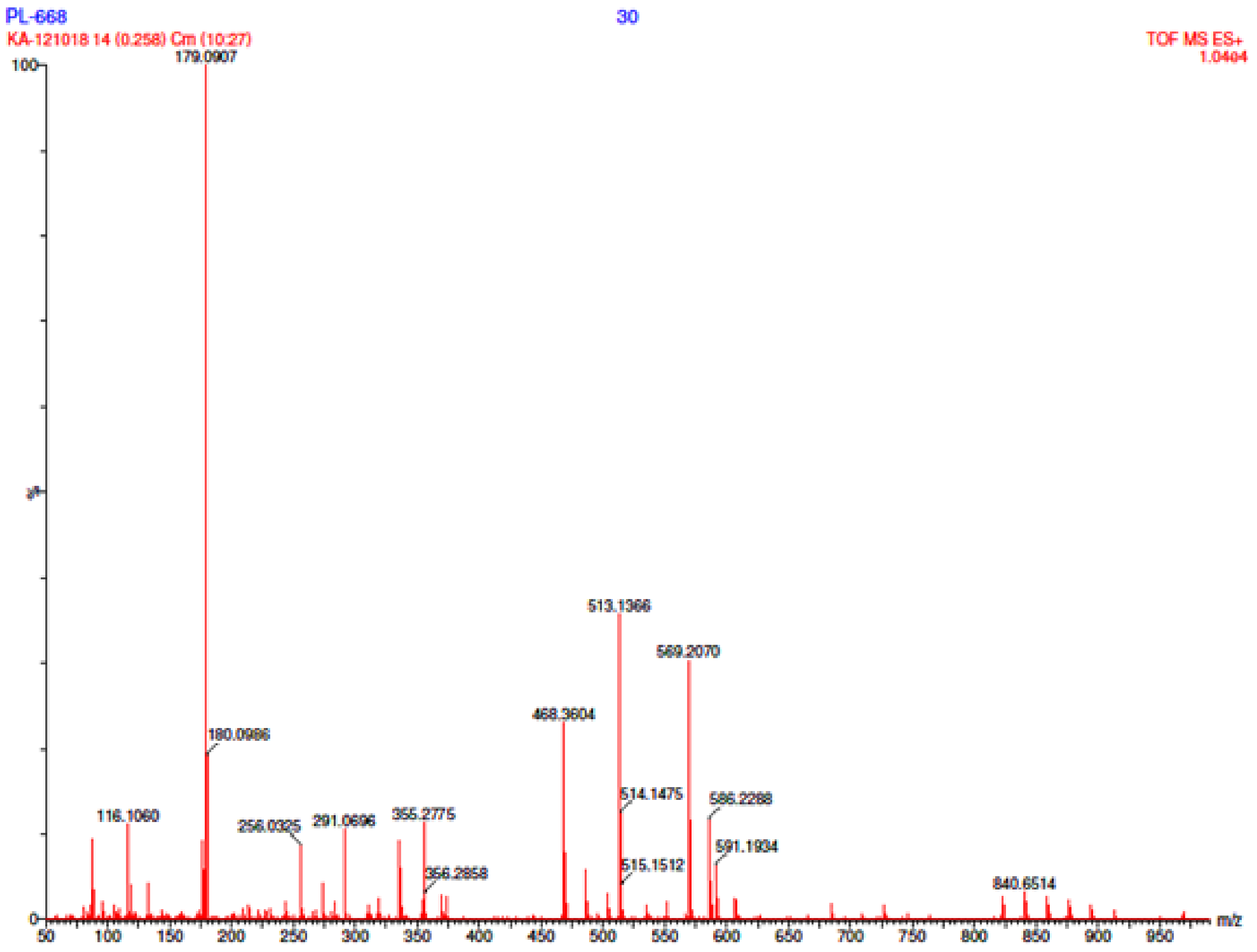

Supplementary Figure 12 Mass spectrum (TOF) of Fmoc-Asp(OtBu)-6-amino-2-cyanobenzothiazole (**4**); 569.2070= $[M+H]^+$

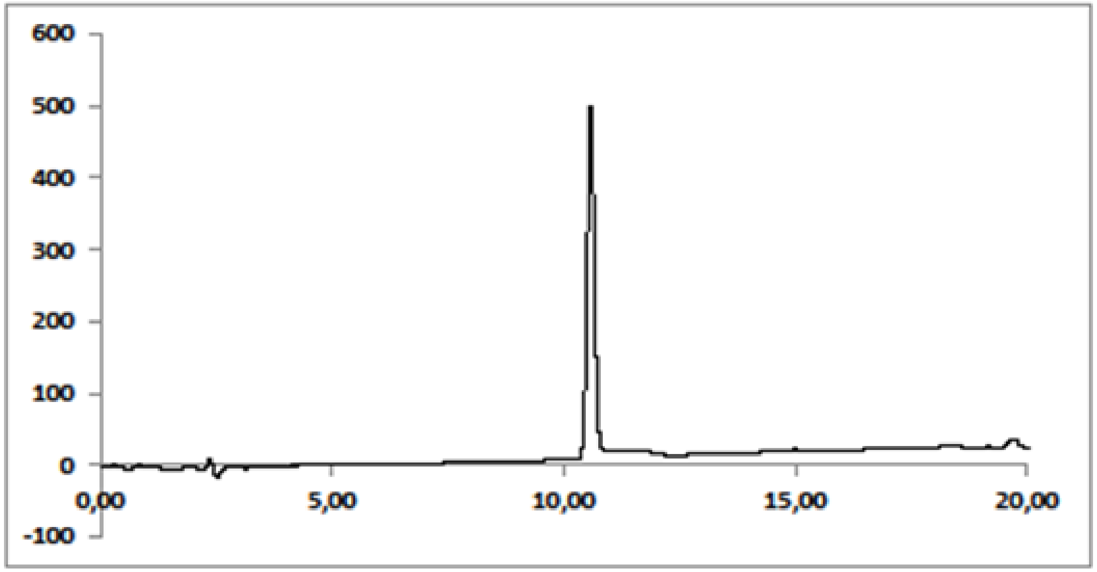

Supplementary Figure 13 RP-HPLC of the purified Fmoc-Asp(OtBu)-6-amino-2-cyanobenzothiazole (**4**); 50-100% B in 25 min, t_R= 10.591 min

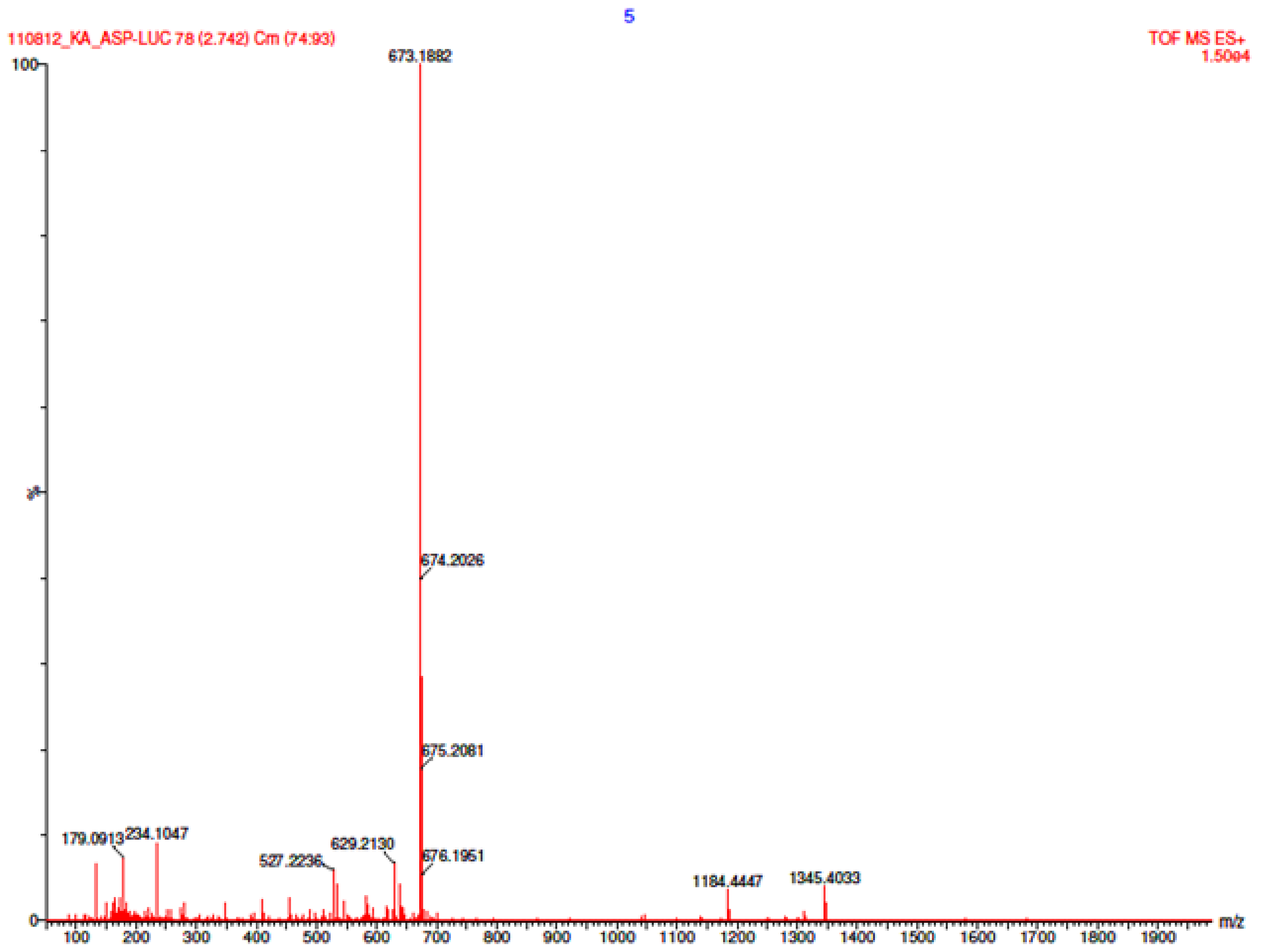

Supplementary Figure 14 Mass spectrum (TOF) of Fmoc-Asp(OtBu)-6-amino-D-luciferin (**5**) 673.1882= $[M+H]^+$

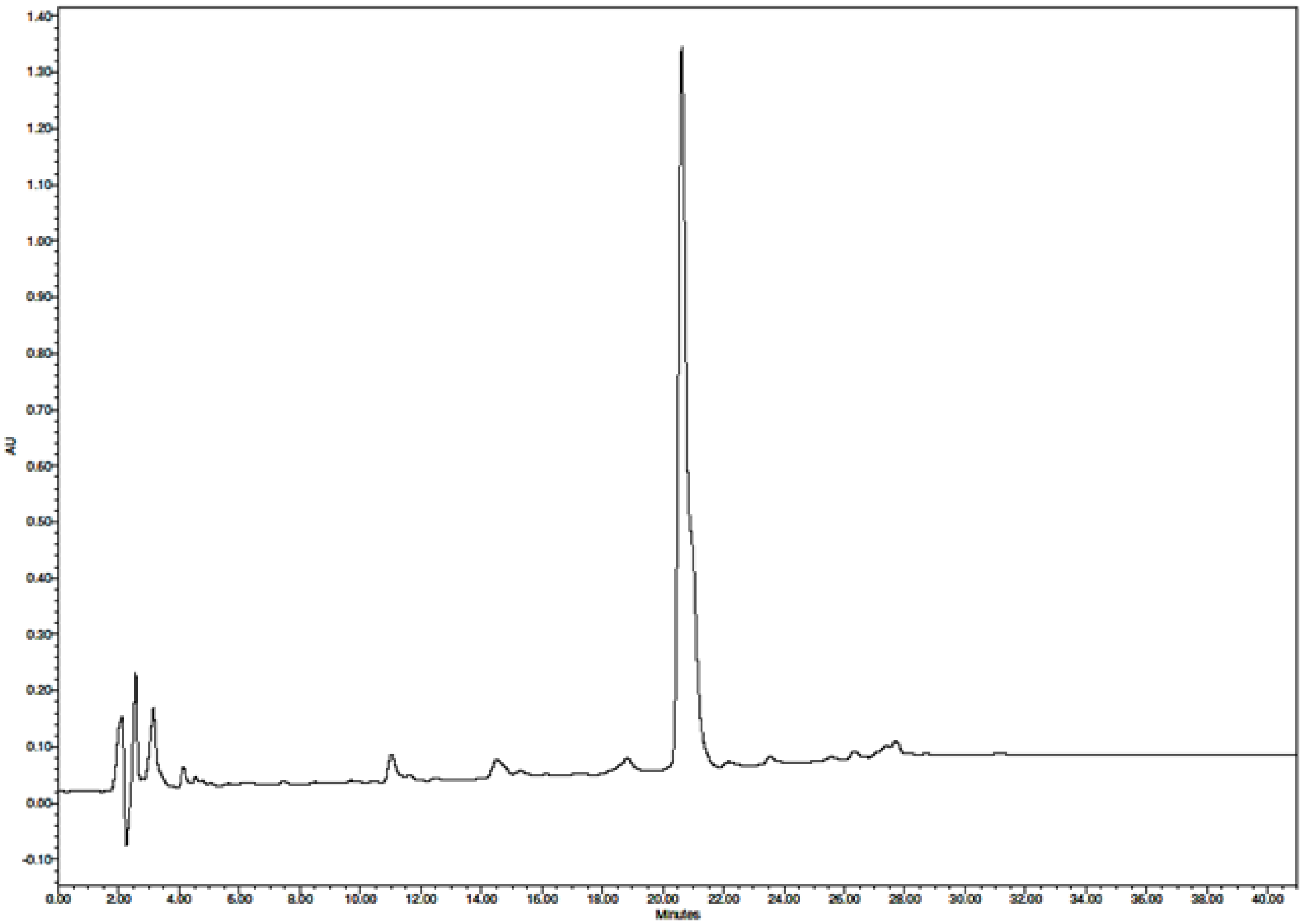

Supplementary Figure 15 RP-HPLC profile of the Fmoc-Asp(OtBu)-6-amino-D-luciferin (**5**) 50-100% B in 25 min, t_R= 21.046 min

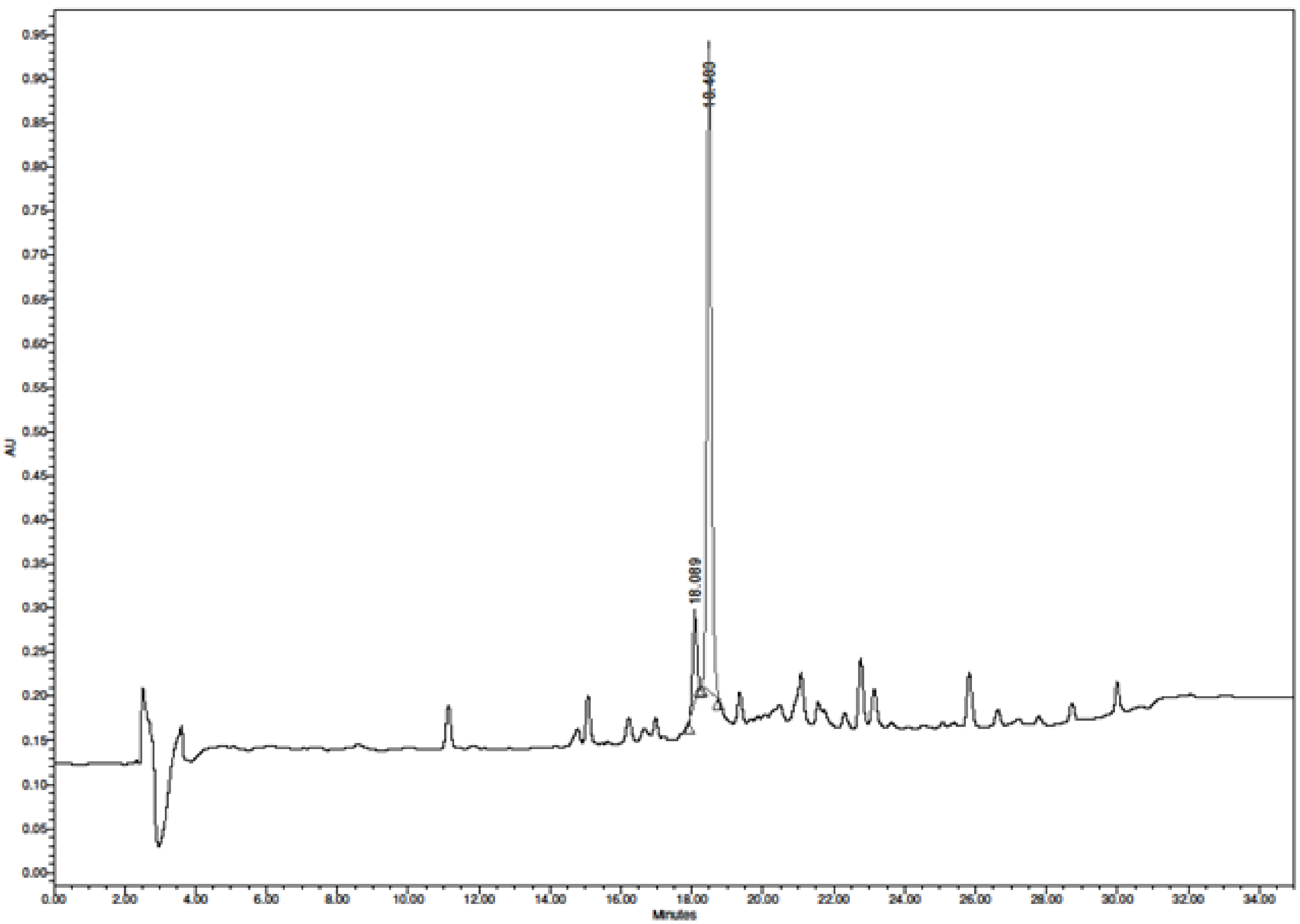

Supplementary Figure 16 RP-HPLC profile of the crude *N*-Z-DEVD-aLuc (**8**)
5-80% B in 25 min + 3 min up to 100% B + 5 min in 100% B
t_R= 18.089 min: *N*-Z-Asp-Glu-Val-Asp-6-amino-L-luciferin
t_R= 18.483 min: *N*-Z-Asp-Glu-Val-Asp-6-amino-D-luciferin

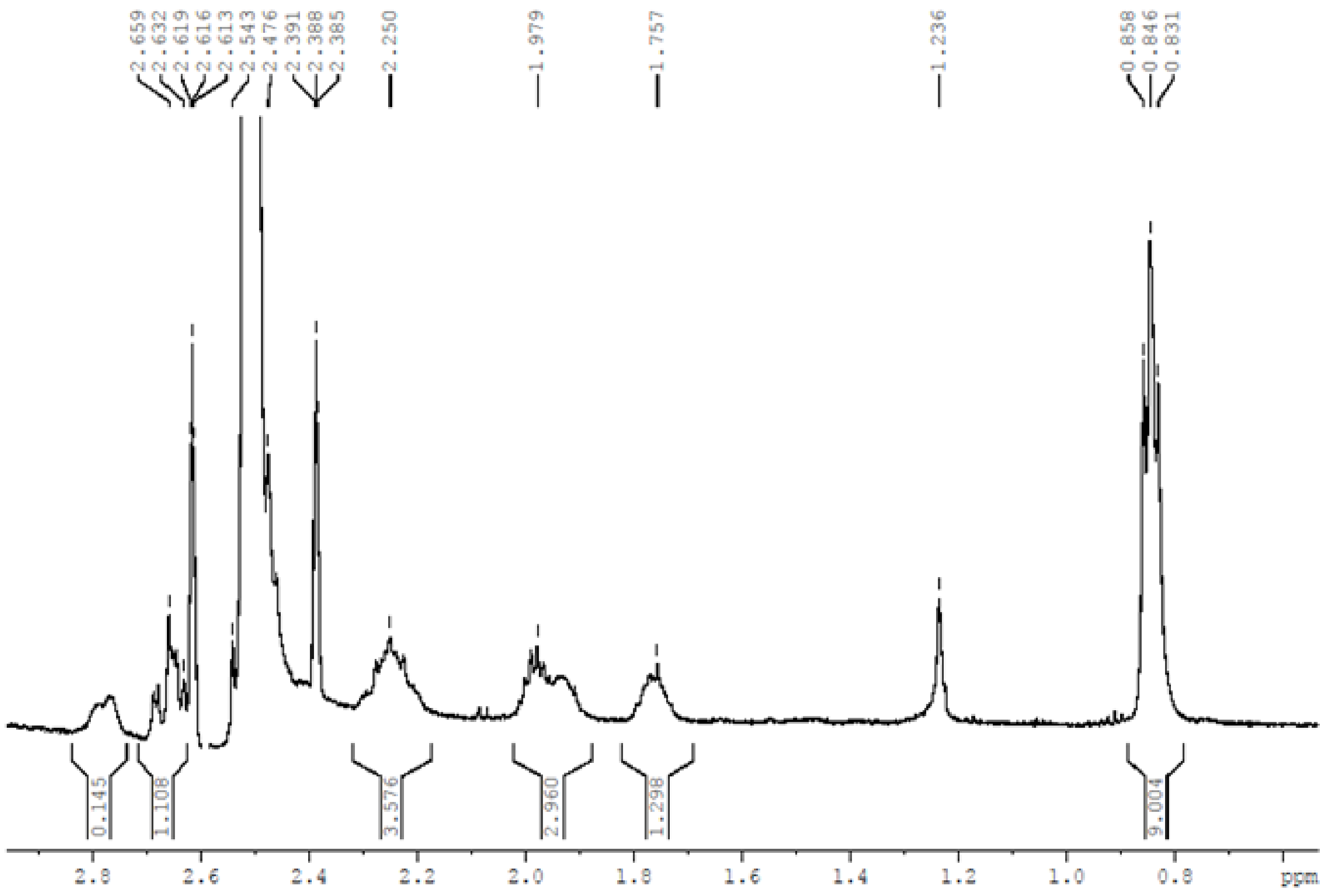

Supplementary Figure 17 part 1 ^{1}H-NMR spectrum of the purified *N*-Z-DEVD-aLuc (**8**)

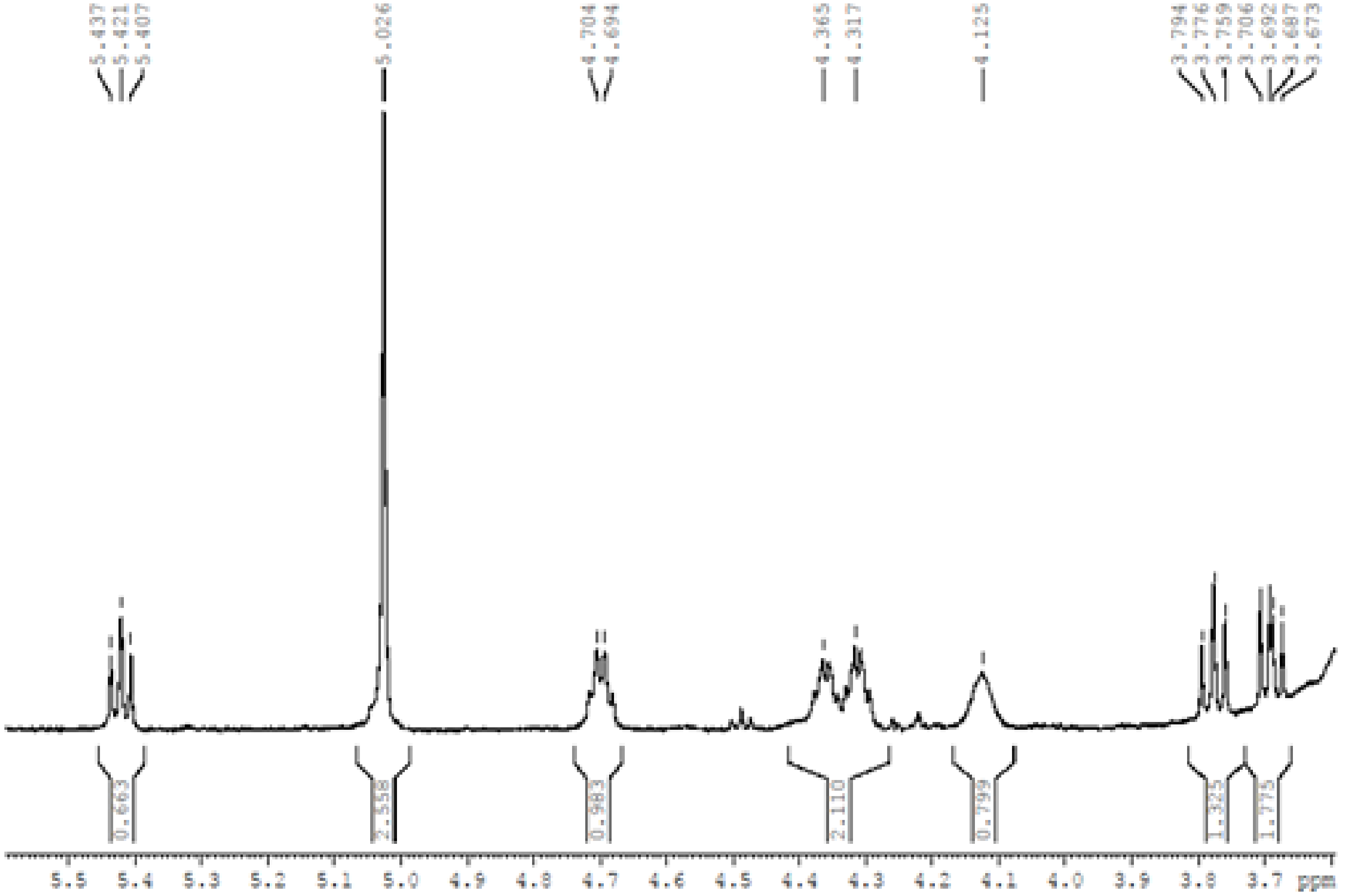

Supplementary Figure 17 part 2 ^{1}H-NMR spectrum of the purified *N*-Z-DEVD-aLuc (**8**)

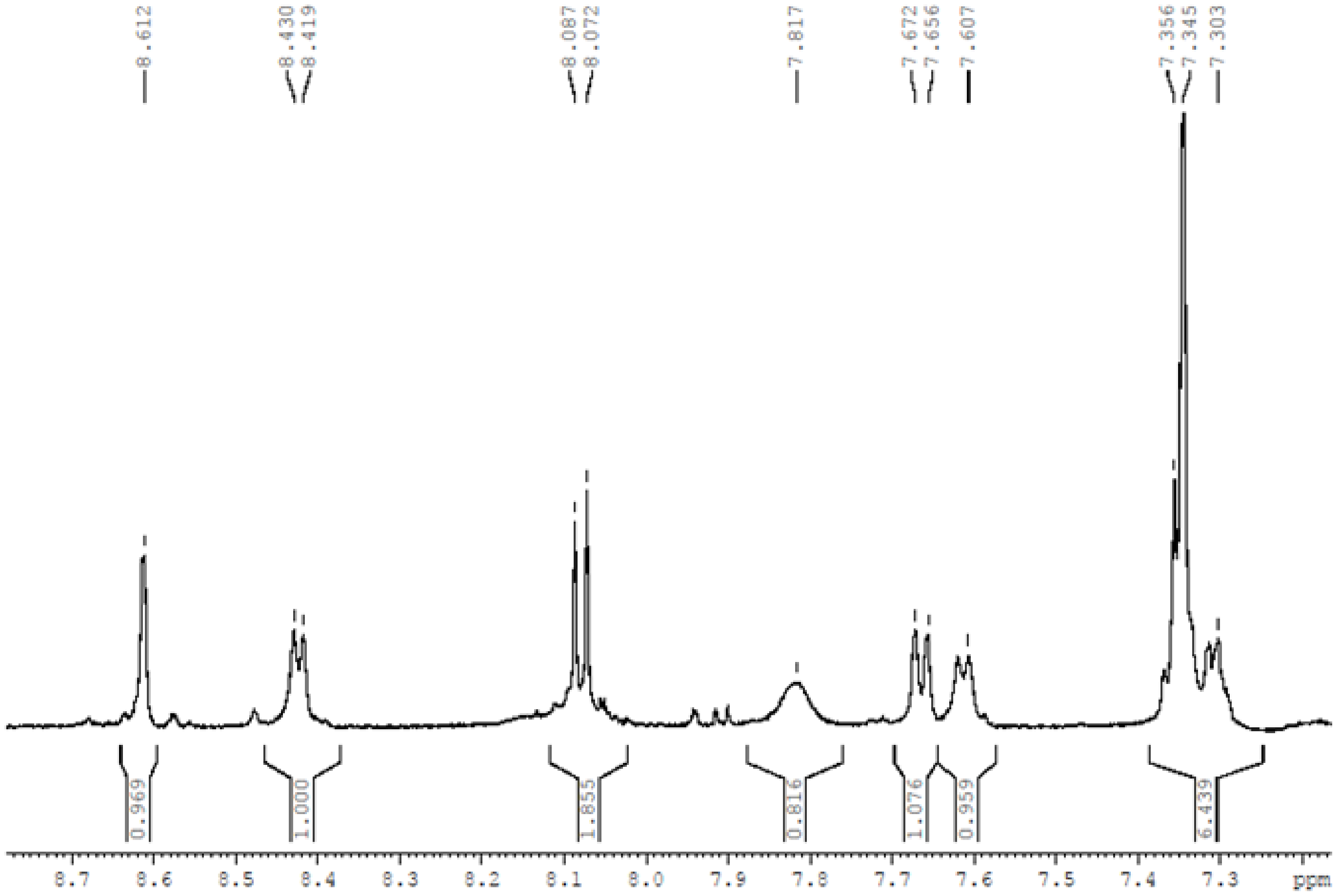

Supplementary Figure 17 part 3 ^{1}H-NMR spectrum of the purified *N*-Z-DEVD-aLuc (**8**)

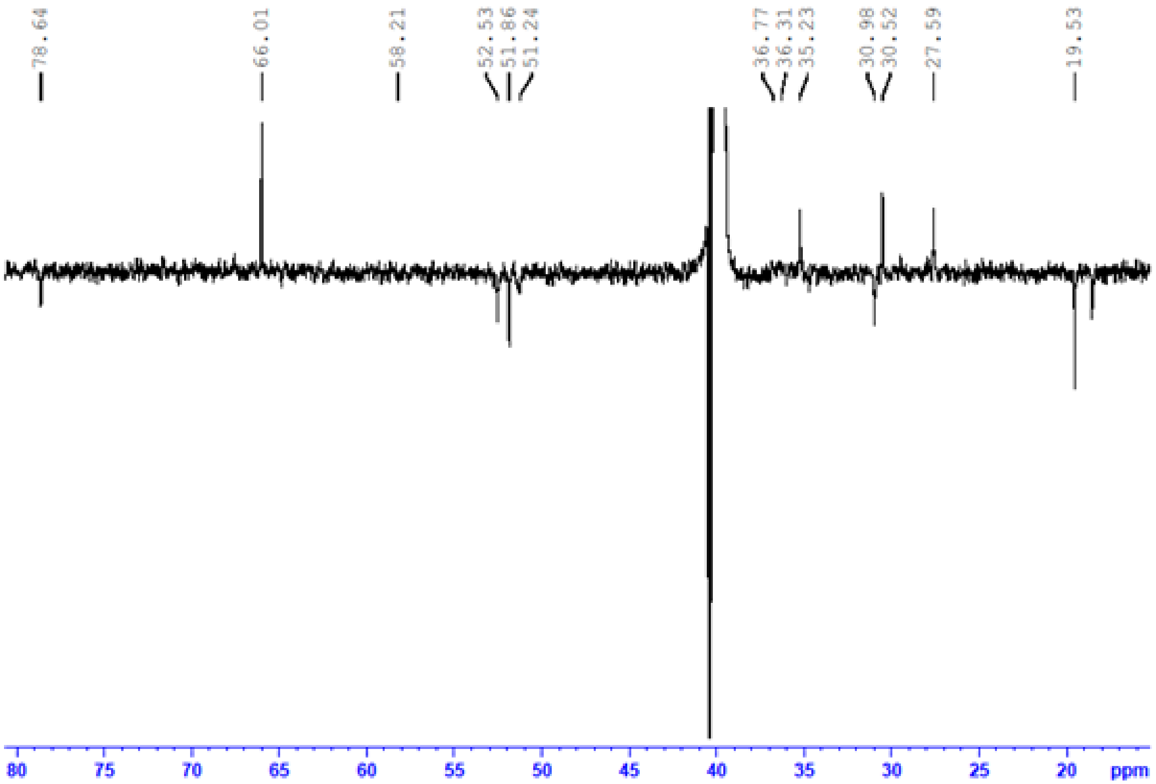

Supplementary Figure 18 part 1 ^{13}C-NMR spectrum of the purified *N*-Z-DEVD-aLuc (**8**)

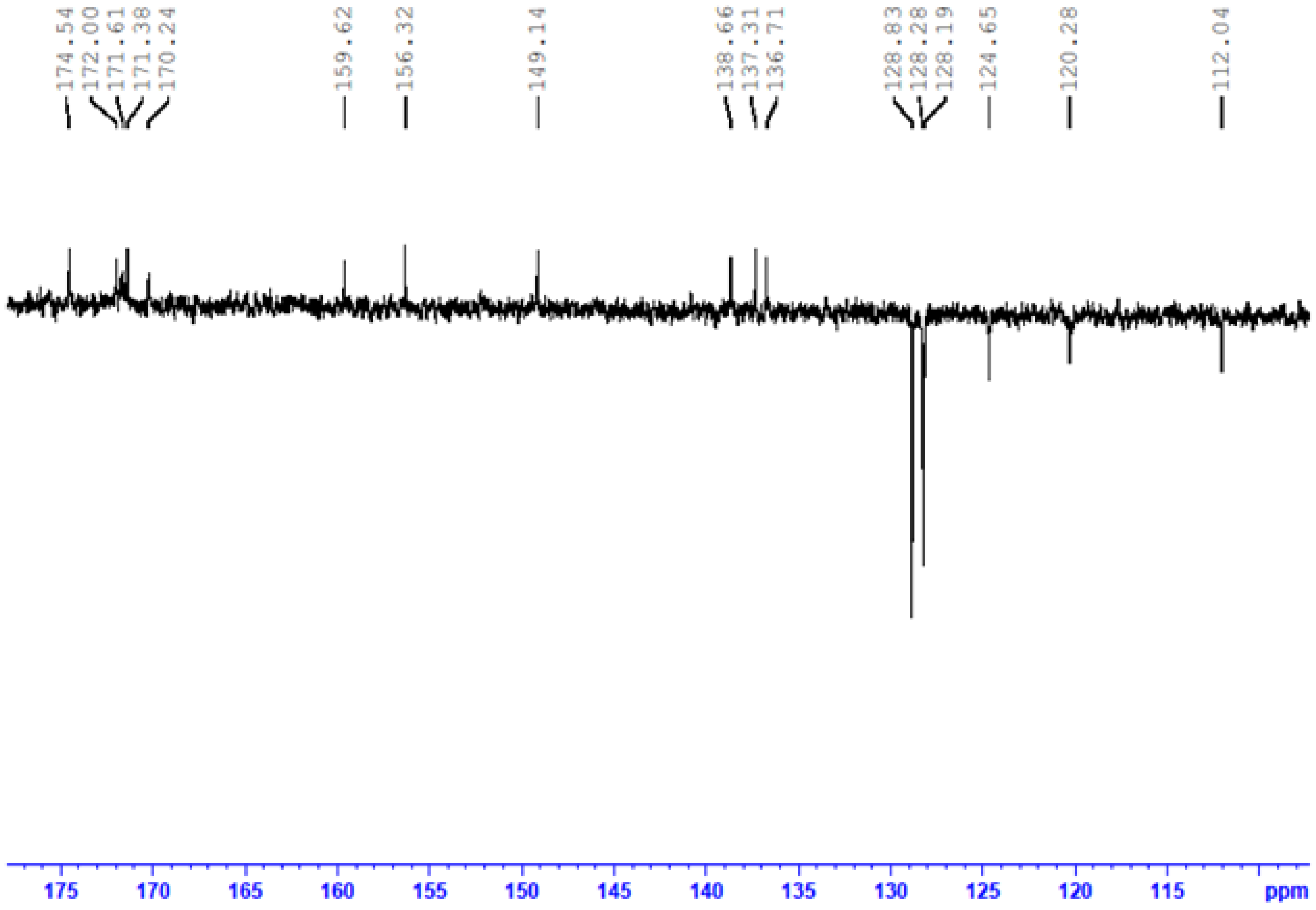

Supplementary Figure 18 part 2 ^{13}C-NMR spectrum of the purified *N*-Z-DEVD-aLuc (**8**)

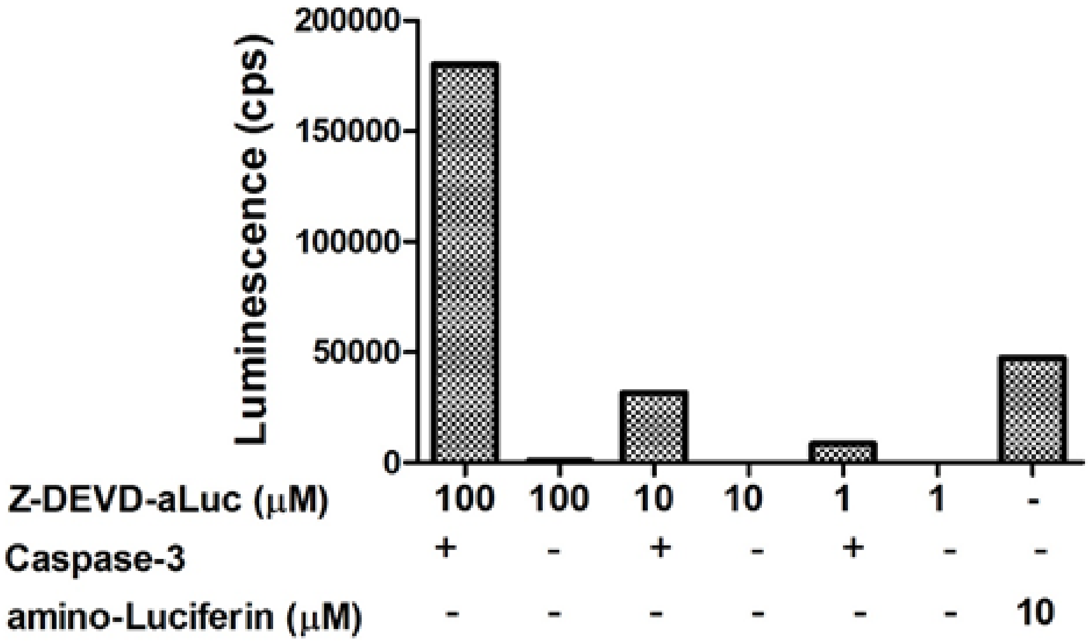

Supplementary Figure 19 The purity of the *N*-Z-DEVD-aLuc peptide is demonstrated. No free aminoluciferin is present in the conjugated peptide, no luminescence background signal could be detected with luciferase enzyme and *N*-Z-DEVD-aLuc even at 0.1 mM concentration. The assay was carried out as described in Supplementary Materials and Methods.

N-ZDEVD-aLuc	100 µM	10 µM	1 µM
**Unit caspase-3:**			
0.227	3663.33	81.19	167.16
0.0227	1432.30	560.17	46.06
0.00227	338.96	133.36	0.00
0.000227	26.15	16.17	7.57
0.0000227	16.17	4.62	2.00

Supplementary Figure 20 The standard error of the mean (SEM) values of Figure 5A. SEM was calculated by Microsoft Excel from triplicate values obtained as described in Materials and Methods.

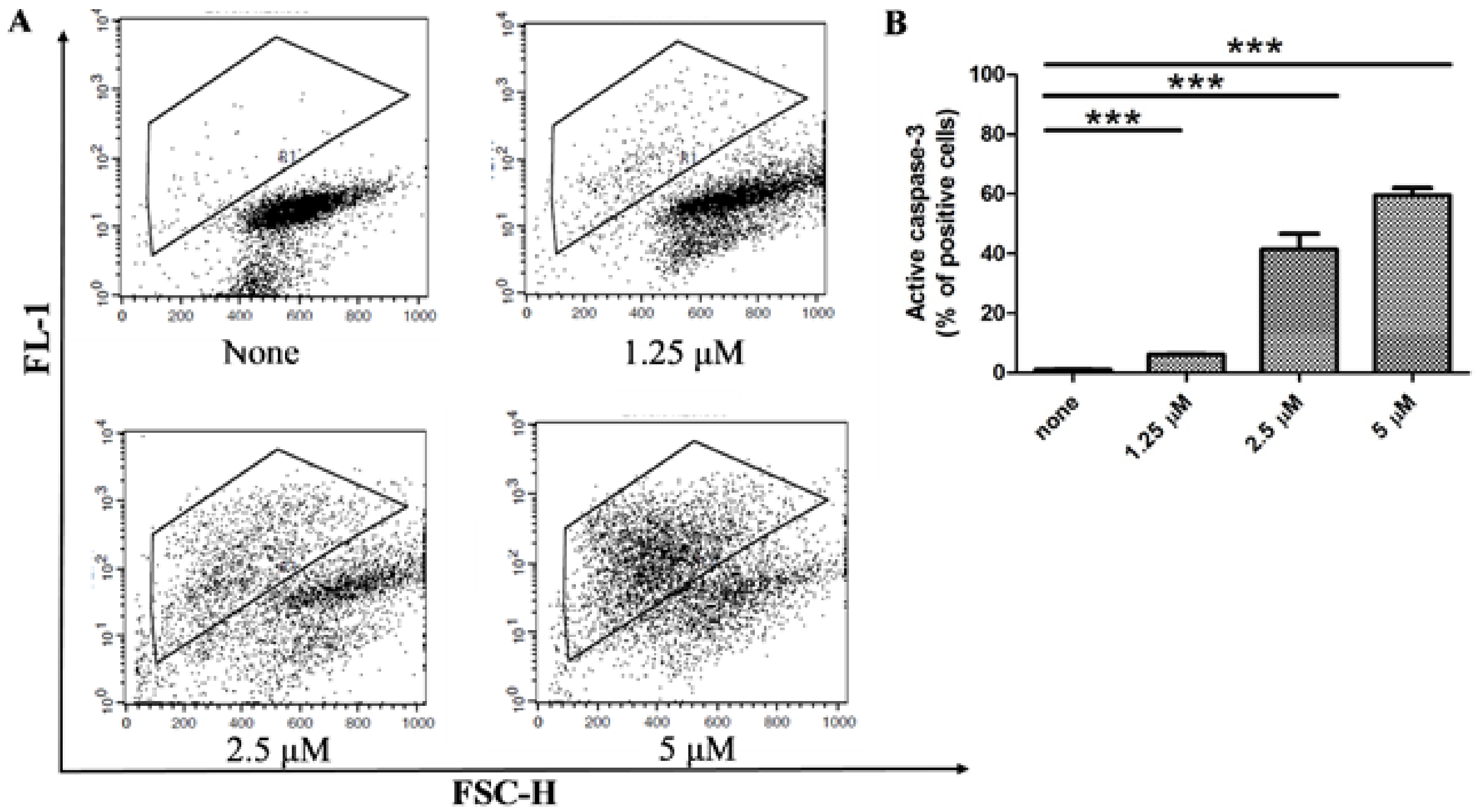

Supplementary Figure 21 The curcumin analogue C150 induces the activation of caspase-3 in A549 cells. (**A**) Representative FL1 - FSC dot plots and (**B**) arithmetic means of percentages ± SEM of cells with active caspase-3 show data of cells treated with curcumin analog C150 with the indicated concentrations (µM) on the graph for 72 h. Active caspase-3 was analyzed by flow cytometry as described in Supplementary Materials and Methods.

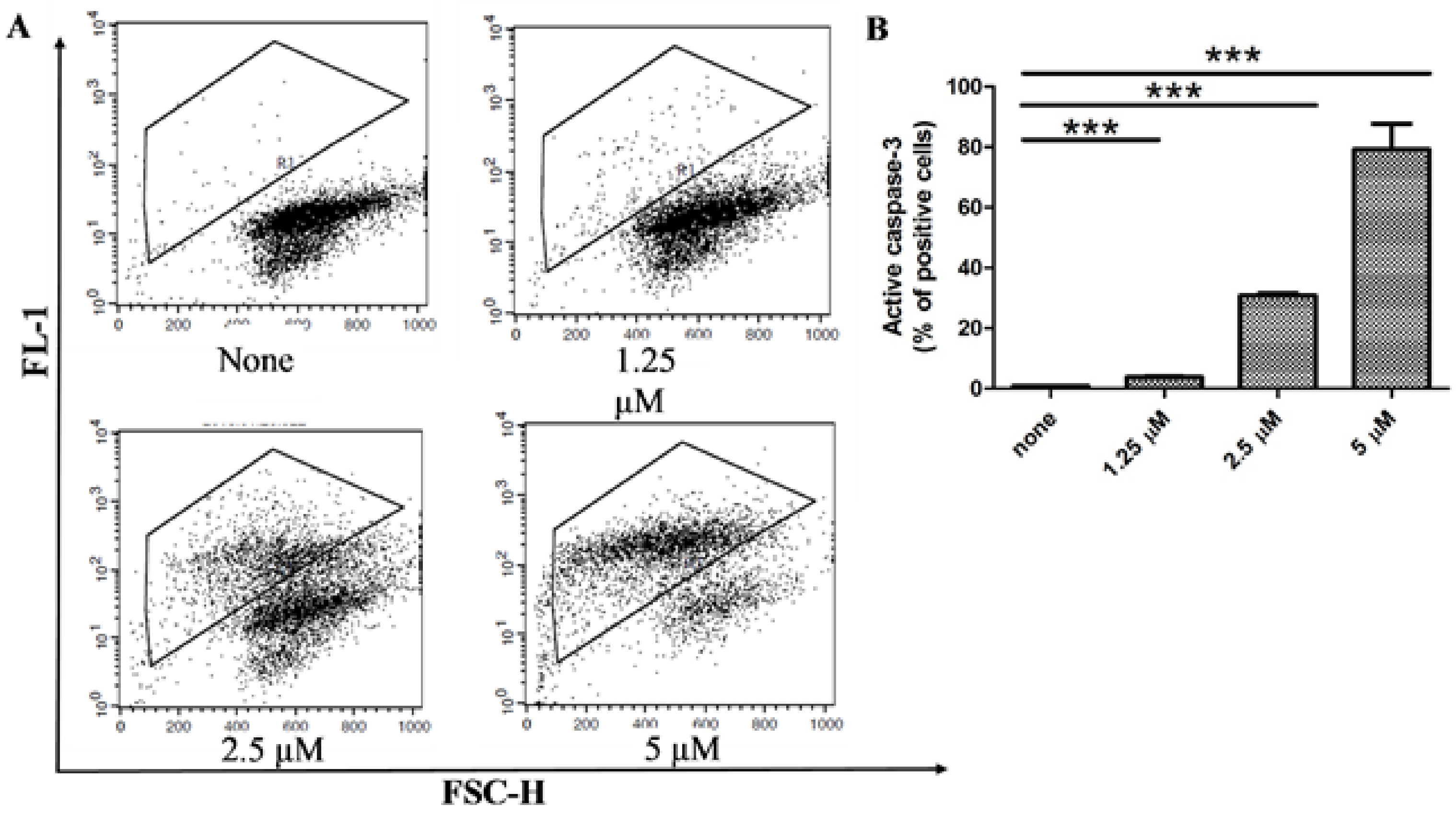

Supplementary Figure 22 Ac-915 induces the activation of caspase-3 in U87 cells. (**A**) Representative FL1 - FSC dot plots and (**B**) arithmetic means of percentages ± SEM of cells with active caspase-3 show data of cells treated with Ac-915 with the indicated concentrations (µM) on the graph for 72 h. Active caspase-3 was analyzed by flow cytometry as described in Supplementary Materials and Methods.

3. Supplementary Materials and Methods

3.1 *In vitro* caspase-3 assay

Caspase-3 and the assay buffer were used from the caspase-3 inhibitor drug screening kit. Caspase-3 was used in 454 mU. *N*-Z-DEVD-aLuc substrate was applied in 100 μM, 10 μM and 1 μM in 50 μl final reaction volume in a black plastic microtiter plate. Positive control aLuc was used in 10 μM. After 90 minutes incubation at 37°C 50 μl luminescence detection reagent was added to each well. Luminescence was recorded as cps by a plate reader within 5 minutes. Blank wells contained each component except caspase-3. Presented values are blank-subtracted.

3.2 Immunofluorescence

Caspase-3 activation was detected by FACS reported previously (Szebeni et al. 2017). Briefly, A549 cells were plated (8 x 10^4) in 24 well tissue culture dishes in 450 μl cell culture media. The day after cells were treated by the curcumin analog C150 in 50 μl in the following concentrations: 5 μM, 2.5 μM, 1.25 μM. After 72 h supernatant was harvested and kept on ice. Cells were washed with PBS and trypsinized (5 minutes, 37°C). Supernatant, washing PBS and media blocked trypsin were mixed and centrifuged down (5 minutes, 4°C, 1800 g). Pellet was resuspended and fixed in 3.5 % PBS buffered formaldehyde for 10 minutes. Cells were washed with FACS-buffer (2 % FCS in PBS), centrifuged (2000 rpm, 5 min). Cells were permeabilized in permeabilization buffer (1 % FCS, 0.1 % saponin in PBS pH 7.4) for 5 minutes. Cells were washed with FACS buffer (2 % FCS in PBS), centrifuged (2000 rpm, 5 min). Rabbit polyclonal anti-cleaved caspase-3 antibody was added at 1:600 dilution in FACS buffer. After incubation for 1h at 4 °C samples were washed two times with FACS buffer. The secondary antibody, polyclonal goat anti-rabbit IgG conjugated with Alexa Fluor® 488 was diluted to 1:600 and incubated with the cells for 30 min at 4 °C. After washing, 300 μl FACS buffer was added for acquisition with the FACSCalibur flow cytometer acquiring 5 x 10^3 events at FL1 channel. Data were analyzed using CellQuest™ software. The percentage of caspase-3 positive cells were gated. Bar graphs were created by GraphPad Prism® 5.
